# Longitudinal Remote SBRT/SRS Training in Latin America: A Prospective Cohort Study

**DOI:** 10.3389/fonc.2022.851849

**Published:** 2022-04-11

**Authors:** Gustavo R. Sarria, Ramsey Timmerman, Michael Hermansen, Sameeksha Malhotra, Betty Chang, Raymond Carter, David A. Martinez, Gustavo J. Sarria, Frank A. Giordano, Indrin J. Chetty, Dante Roa, Benjamin Li

**Affiliations:** ^1^ Rayos Contra Cancer, Inc., Nashville, TN, United States; ^2^ Department of Radiation Oncology, University Hospital Bonn, University of Bonn, Bonn, Germany; ^3^ Northwestern University Feinberg School of Medicine, Chicago, IL, United States; ^4^ Department of Radiation Oncology, Banner MD Anderson Cancer Center, Gilbert, AZ, United States; ^5^ Vanderbilt University, Nashville, TN, United States; ^6^ University of Illinois College of Medicine, Chicago, IL, United States; ^7^ Molecular Oncology Division, St. Jude Children’s Research Hospital, Memphis, TN, United States; ^8^ Department of Radiation Oncology, Oncosalud-Auna, Lima, Peru; ^9^ Department of Radiotherapy, Instituto Nacional de Enfermedades Neoplasicas, Lima, Peru; ^10^ Department of Radiation Oncology, Henry Ford Cancer Institute, Henry Ford Health System, Detroit, MI, United States; ^11^ Department of Radiation Oncology, University of California Irvine, Orange, CA, United States; ^12^ Department of Radiation Oncology, University of California San Francisco, San Francisco, CA, United States

**Keywords:** telehealth, SBRT, SRS, continuing medical education, global health

## Abstract

**Background:**

Continuing medical education in stereotactic technology are scarcely accessible in developing countries. We report the results of upscaling a longitudinal telehealth training course on stereotactic body radiation therapy (SBRT) and stereotactic radiosurgery (SRS), after successfully developing a pilot course in Latin America.

**Methods:**

Longitudinal training on SBRT and SRS was provided to radiation oncology practitioners in Peru and Colombia at no cost. The program included sixteen weekly 1-hour live conferencing sessions with interactive didactics and a cloud-based platform for case-based learning. Participant-reported confidence was measured in 16 SBRT/SRS practical domains, based on a 1-to-5 Likert scale. Pre- and post-curriculum exams were required for participation credit. Knowledge-baseline, pre- and post-curriculum surveys, overall and single professional-group confidence changes, and exam results were assessed.

**Results:**

One hundred and seventy-three radiotherapy professionals participated. An average of 56 (SD ±18) attendees per session were registered. Fifty (29.7%) participants completed the pre- and post-curriculum surveys, of which 30% were radiation oncologists (RO), 26% radiation therapists (RTT), 20% residents, 18% medical physicists and 6% neurosurgeons. Significant improvements were found across all 16 domains with overall mean +0.55 (SD ±0.17, p<0.001) Likert-scale points. Significant improvements in individual competences were most common among medical physicists, RTT and residents. Pre- and post-curriculum exams yielded a mean 16.15/30 (53.8 ± 20.3%) and 23.6/30 (78.7 ± 19.3%) correct answers (p<0.001).

**Conclusion:**

Longitudinal telehealth training is an effective method for improving confidence and knowledge on SBRT/SRS amongst professionals. Remote continuing medical education should be widely adopted in lower-middle income countries.

## Introduction

Access to continuing medical education in radiation oncology remains a major obstacle in low- and middle-income countries (LMICs) (1, 2). Rayos Contra Cancer (RCC - Rayos Contra Cancer Inc. Vanderbilt, TN, USA), a non-profit organization founded in 2018, has focused on bridging this gap in developing countries around the globe (3). For LMICs to keep pace with current trends in radiation therapy (RT), education in stereotactic body radiation therapy (SBRT) and stereotactic radiosurgery (SRS) is essential. A small investment in education and training can yield major benefits by improving clinical treatment options and patient throughput at facilities that are past capacity (4) which often times is the reality in these countries.

To this effect, many countries, including some in Latin America (Latam), have invested in technology upgrades for SBRT/SRS in the last two decades. However, full implementation of these techniques remains poorly adopted due to several factors, including lack of expertise (5–7). Hence, there is an urgent need to improve SBRT/SRS utilization rates, improve healthcare provision, and diminish patient financial toxicity (8).

In 2019, RCC successfully developed a pilot study to assess and compare the benefits of remote SBRT/SRS longitudinal training against single-time on-site lecturing. The data showed the superiority of longitudinal training which motivated RCC to expand these remote training curricula to different latitudes (9). Helping to bring experienced international educators and LMIC practitioners closer may improve knowledge in specific subject areas, boost confidence in delivering the highest quality treatment, and improve clinical outcomes of all patients receiving treatment (10).

Herein, we report the outcomes of an international SBRT/SRS telehealth training course carried out in Latam, comparing self-confidence and knowledge pre- and post-curriculum for applying SBRT/SRS treatment modalities in daily practice.

## Materials and Methods

### Study Development and Intervention

A remote training course was planned for six RT centers in Peru and Colombia, which had recently acquired or planned to acquire SBRT and SRS treatment technology. Each center received an initial course debriefing (4 on-site and 2 on-line) and subsequent telehealth weekly sessions. Based on demand, enrollment was granted to further centers in the region without on-site debriefing. Multi-disciplinary RT teams - including medical physicists, radiation oncologists, residents, and radiation therapists - were invited to join. All participants were required to fill a pre-curriculum survey with demographic questions, measured baseline SBRT/SRS knowledge, and application confidence levels. Thereafter, a 30-question knowledge-based exam were given to the participants without disclosure of the answers.

The course format was designed by RCC and consisted of weekly sessions between January and April 2020, modeled according to the Project Extension for Community Healthcare Outcomes (ECHO) model (ECHO Institute, New Mexico, USA) (6, 11). A team of volunteer faculty with expertise in clinical and technical aspects of SBRT and SRS developed the aforementioned 30-question multiple-choice exam encompassing different RT-related concepts. These educators delivered sixteen 1-hour educational sessions *via* live video conferencing, which covered practical elements of small-field physics, radiobiology, radiotherapy linear accelerator (linac) commissioning and quality assurance (QA), image guidance, motion management, treatment planning, dosimetry, and disease-site specific clinical training. The cloud-based platform, ProKnow DS™ (ProKnow, Sanford, FL, USA), was used for RT case-based learning. Lectures and links to recorded sessions are detailed in **Appendix 1** (see **Supplementary Material**).

Upon completion of the course, participants were asked to fill a post-curriculum survey and repeat the initial exam. Self-confidence levels regarding SBRT/SRS knowledge and application were measured on a 1-5 Likert scale. The collected information was stored in the RedCap system (Research Electronic Data Capture, Vanderbilt University, Tennessee, USA) (6, 11). The detailed survey and exam items are provided in **Appendix 2** (see **Supplementary Material**) in both Spanish and English.

### Statistical Analysis and Endpoints

The overall mean changes are shown for participants who completed both pre- and post-curriculum surveys. The statistical significance was analyzed for changes between both surveys and attendance rates for pre-curriculum non-debriefed (nD) and debriefed (D) facilities according to the Analysis of Variance (ANOVA) method. Pre- and post-curriculum exam results for the entire cohort and confidence level sub-analysis for each professional sub-group were performed through the t-test for quantitative, discrete variables. All statistical analysis was performed with The jamovi project (2020). *jamovi*. (Version 1.2) [Computer Software]. Retrieved from https://www.jamovi.org. A statistically significant change was established at the p<0.05 significance level.

### Ethics Statement

This research was performed according to the principles of the Declaration of Helsinki. All participants’ information is confidential and data was anonymized prior to analysis. The study was released from institutional review board (IRB) approval due to its non-clinical nature.

## Results

One hundred and seventy-three attendees from 19 RT centers in Peru (n=16) and Colombia (n=3), participated in the course. One hundred and sixty-eight participants completed the pre-curriculum survey, which comprised 70 (41.7%) radiation oncologists, 46 (27.4%) radiation therapists, 25 (14.9%) medical physicists, 24 (14.3%) residents and 3 (1.8%) neurosurgeons. No differences were found between participants’ prior sources of SBRT/SRS training (p=0.789), including 23.2% and 23.8% of participants who declared not having received previous training, 15.5% and 11.3% access to on-line education, and 43.5% and 42.3% self-teaching for SBRT and SRS, respectively. Further details are reported in **Table 1**.

**Table 1:** Reported sources of training.

Sources of training	SRS	SBRT
	*n=*168	
No training	23.8%	23.2%
Online Modules	11.3%	15.5%
Independent Study	42.3%	43.5%
In-person conferences/workshops	39.9%	38.7%
Industry/manufacturers	20.2%	17.9%
Formal/certified academic programs	25.0%	25.0%

SRS, stereotactic radio-surgery; SBRT, stereotactic body radiotherapy. Results from the multiple-choice questionnaire, showing prior sources of training. No significant differences between SRS and SBRT training access were found (p = 0.789).

A mean 56 ( ± 18) attendees per session were registered. When assessed per groups, D facilities (*n*=6) included 116 registered participants, yielding a total possible of 1972 individual attendances of which 835 (42.3%) were achieved; the nD (*n*=13) facilities accounted for 57 registered attendants, yielding a total possible of 969 individual attendances, of which 202 (20.8%) were achieved. A significant statistical difference in attendance was observed favoring group D (p<0.0001). Competences and corresponding baseline confidence level of the entire cohort are given in **Table 2**.

**Table 2:** Baseline self-confidence levels.

Question items	Baseline Likert-scale scores (%), *n=*168					Mean values (1-5)
	1	2	3	4	5	
1) I am confident in managing SBRT cases	4	7	32	45	13	3.6
2) I am confident in managing SRS cases	4	6	26	48	17	3.7
3) What is your current familiarity to run a SBRT program?	5	38	45	11	2	2.7
4) What is your current familiarity to run a SRS program?	5	35	42	15	2	2.7
5) What is your current ability to teach another physicist how to safely use SBRT?	29	31	32	8	1	2.2
6) What is your current ability to teach another physicist how to safely use SRS?	28	30	32	10	0	2.2
7) What is your current confidence to fully implement a SBRT/SRS program from the beginning to the end?	18	31	33	17	1	2.5
8) What is your current confidence in understanding SBRT/SRS physics?	11	35	32	20	3	2.7
9) What is your current confidence in understanding the simulation and movement control processes in SBRT/SRS?	7	23	39	26	5	3.0
10) What is your current confidence in reviewing SBRT/SRS dosimetry?	14	27	29	17	3	2.7
11) What is your current confidence in generating SBRT/SRS plans?	21	26	34	18	2	2.5
12) What is your current confidence in understanding the importance of IGRT for SBRT/SRS?	6	23	33	29	9	3.1
13) What is your current confidence in understanding the infrastructure, hardware and software requirements for SBRT/SRS?	11	26	34	21	8	2.9
14) What is your current confidence in understanding SBRT/SRS commissioning?	16	33	34	14	2	2.5
15) What is your current confidence in understanding the quality assurance process for SBRT/SRS?	17	27	36	17	3	2.6
16) What is your current confidence in understanding the clinical applications of SBRT/SRS?	7	18	39	30	6	3.1

SRS, stereotactic radio-surgery; SBRT, stereotactic body radiotherapy; IGRT, image-guided radiotherapy.

Liker-scale categories correspond as follows:

Questions 1–2: (1: strongly disagree, 2: disagree, 3: neutral, 4: agree and 5: strongly agree).

Questions 3-4: (1: no familiarity; 2: little familiarity; 3: medium familiarity; 4: significant familiarity; 5: expert familiarity).

Questions 5-6: (1: no ability; 2: little ability; 3: medium ability; 4: significant ability; 5: expert ability).

Questions 7-16: (1 = not confident, 2 = a little confident, 3 = moderately confident, 4 = very confident y 5 = extremely confident).

Overall mean values for the entire cohort are displayed on the last column.

Of the 168 initial survey responders, 50 (29.8%) participants completed both pre- and post-curriculum surveys, of which 30% (*n=*15) were ROs, 26% (*n=*13) RTTs, 20% (*n=*10) residents, 18% (*n=*9) medical physicists and 6% (*n=*3) neurosurgeons. Overall self-confidence and knowledge levels for performing SBRT and SRS improved a mean 0.55 ( ± 0.17, p<0.001) Likert-scale points. When assessing each questionnaire’s item, significant improvements were found in self-confidence and competence levels for medical physicists, radiation therapists, and residents. Mean confidence level changes and statistical values for individual professional groups and questions are shown in **Table 3**.

**Table 3:** Confidence level dynamics after remote training.

Question items	Mean confidence level changes/p= values					
	RO	RTT	Residents	Physicists	NS	All
1) I am confident in managing SBRT cases	0.12	0.15	-0.1	0.42	0.33	0.14
2) I am confident in managing SRS cases	0.19	0.15	-0.2	0.42	0.67	0.16
3) What is your current familiarity to run a SBRT program?	0.53	0.38	**1.0**	0.57	1.0	0.62
			**0.004**			
4) What is your current familiarity to run a SRS program?	0.4	0.38	**0.9**	0.67	0.67	0.56
			**0.01**			
5) What is your current ability to teach another physicist how to safely use SBRT?	0.41	**0.92**	**0.60**	0.34	0.33	0.58
		**0.004**	**0.024**			
6) What is your current ability to teach another physicist how to safely use SRS?	0.35	**0.92**	**0.60**	0.24	0.33	0.54
		**0.004**	**0.024**			
7) What is your current confidence to fully implement a SBRT/SRS program from the beginning to the end?	0.45	**1.08**	0.2	0.56	0	0.58
		**0.003**				
8) What is your current confidence in understanding SBRT/SRS physics?	0.46	0.77	0.2	0.69	-0.67	0.50
9)What is your current confidence in understanding the simulation and movement control processes in SBRT/SRS?	0.62	**1.0**	0.3	**0.7**	0	0.68
		**0.009**		**0.05**		
10) What is your current confidence in reviewing SBRT/SRS dosimetry?	0.64	0.69	-0.2	0.59	0.33	0.52
11) What is your current confidence in generating SBRT/SRS plans?	0.65	**0.85**	0.1	**0.82**	-0.33	0.62
		**0.005**		**0.023**		
12) What is your current confidence in understanding the importance of IGRT for SBRT/SRS?	0.6	**1.0**	0.2	**1.22**	0.33	0.78
		**0.016**		**0.013**		
13) What is your current confidence in understanding the infrastructure, hardware and software requirements for SBRT/SRS?	0.56	**1.08**	0	**0.91**	0	0.68
		**0.024**		**0.023**		
14) What is your current confidence in understanding SBRT/SRS commissioning?	0.74	0.54	-0.1	**0.91**	0.33	0.56
				**0.002**		
15) What is your current confidence in understanding the quality assurance process for SBRT/SRS?	0.7	**0.77**	0	**0.91**	0.33	0.62
		**0.006**		**0.002**		
16) What is your current confidence in understanding the clinical applications of SBRT/SRS?	0.71	0.62	0.3	**0.89**	-0.33	0.64
				**0.023**		
Statistical significance value (p=) per professional group	**<0.001**	**<0.001**	**0.021**	**<0.001**	0.065	**<0.001**

SRS, stereotactic radio-surgery; SBRT, stereotactic body radiotherapy; IGRT, image-guided radiotherapy; RO, radiation oncologist; RTT, radiation therapists; NS, neurosurgeon. Mean confidence level changes per professional group and statistical significance value. Individual questions and professional groups show statistical significance when positive.

Liker-scale categories correspond as follows:

Questions 1–2: (1: strongly disagree, 2: disagree, 3: neutral, 4: agree and 5: strongly agree).

Questions 3-4: (1: no familiarity; 2: little familiarity; 3: medium familiarity; 4: significant familiarity; 5: expert familiarity).

Questions 5-6: (1: no ability; 2: little ability; 3: medium ability; 4: significant ability; 5: expert ability).

Questions 7-16: (1 = not confident, 2 = a little confident, 3 = moderately confident, 4 = very confident y 5 = extremely confident).

Overall mean values for the entire cohort are displayed on the last column.

Bold values are statistically significant.

**Figure 1:**
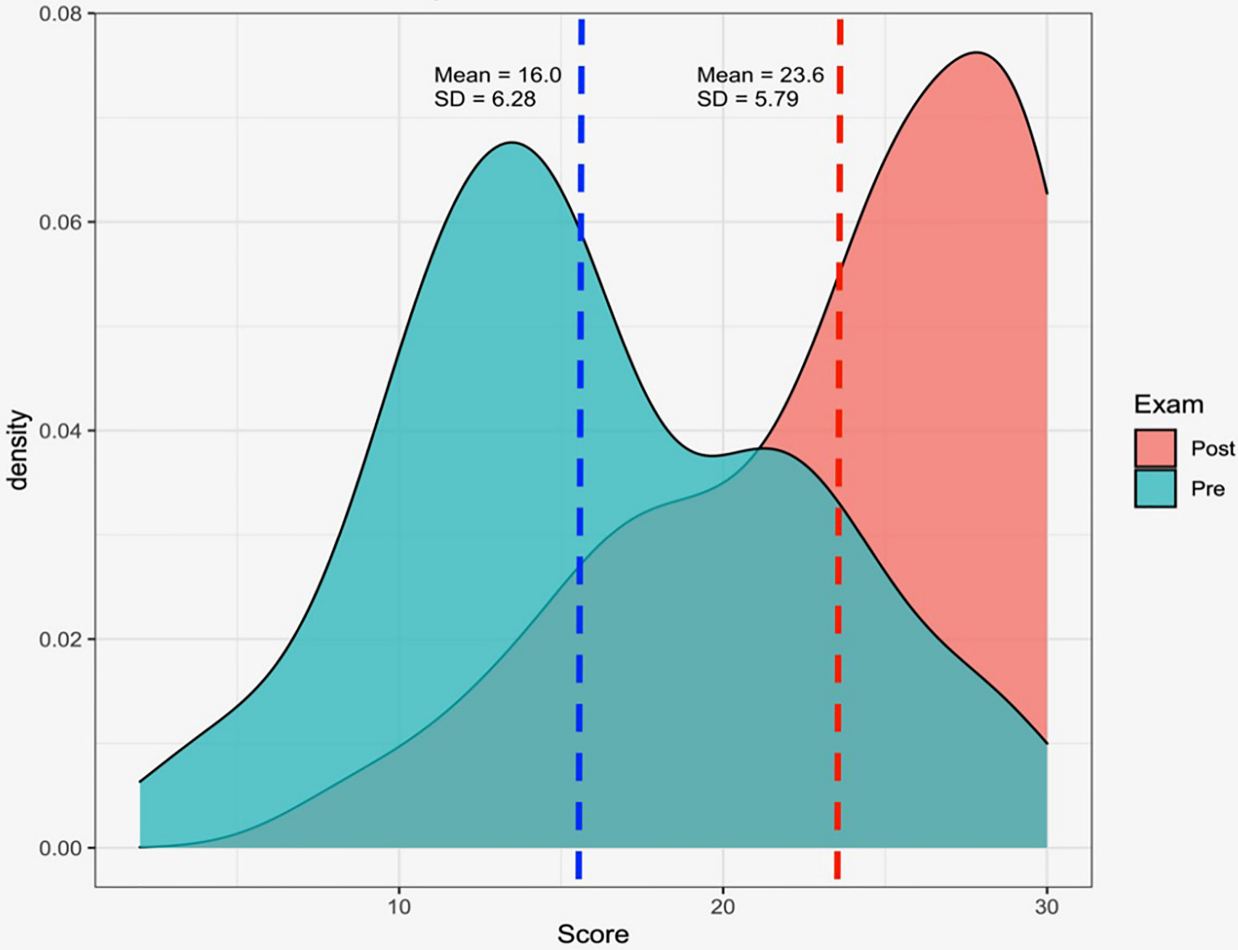
Scoring tendencies for pre- and post-curriculum test. Pre- and post-curriculum raw scoring, showing a clear trend towards improved exam scoring after longitudinal lecturing.

One hundred and forty-one participants completed the pre-curriculum exams, scoring a mean 16.15 ( ± 6.09) correct answers (53.8%) out of a 30 total possible. After curriculum completion, 81 participants fulfilled the exam, with a mean 23.6 ( ± 5.78) correct answers (78.7%), yielding a statistical significant difference between both tests (p<0.001). Scoring frequencies and distributions are shown in **Figure 1**. The detailed question-by-question scoring for both pre- and post-curriculum tests can be found in **Appendix 3** (see **Supplementary Material**).

## Discussion

SRS and SBRT are cornerstone components of modern treatment strategies in radiation oncology. Many scientific studies and prospective clinical trials have proven their role in both curative and palliative intents. However, despite these benefits (12), shifting practice standards to SRS/SBRT has many challenges manifested in clinical and technological training, and expertise (13). Developed countries have already experienced this migration and have been benefitting from this technology for decades both clinically and financially. Furthermore, the ongoing pandemic has propelled an increase in hypofractionation utilization rates in different latitudes, thereby creating more opportunities for establishing this approach as the future standard (14).

n LMICs, however, the lack of educational sources and formal training, technical expertise, and inadequate compensation, have limited the implementation of SRS and SBRT. According to a recently published survey study by *Rodin* et al., the most common practitioner-reported barriers in Latam are insufficient technology (24.2%) and reimbursement (14.3%). On the other hand, reimbursement was reported as a motive for hypofractionation in 5.4% of cases, while a statistically significant relationship was found for hypofractionation underutilization in LMICs in Latam and Asia-Pacific. Moreover, it should be noted that IMRT availability in the region was reported at 77.5% of centers and university affiliation at 36.1% of those, while scope of practice was public at 43.5%, private at 60% and public-private at 27% from a total 285 regional responders. Interestingly, the lack of long-term data (36.3%) and concerns regarding acute (23.7%) and late toxicity (29.1%) were reported as reasons for not adopting hypofractionation in daily practice. Conversely, opportunities were highlighted as resource optimization for improved machine availability and lower costs in 70.5% and 53.9%, respectively (8).

When strictly focusing on academic-related issues, a potential effect on 30 – 40% of decision-making could be inferred by enforcing academic approaches. Moreover, framed in the ongoing COVID-19 pandemic, telehealth solutions are able to bring patients and practitioners closer (15). This initiative could be additionally translated to continuing medical education, as the current growth of telehealth is cementing a post-COVID era with increased web-based interactivity (16).

Initial evidence provided by a pilot study led by our group showed that longitudinal web-based education might carry increased learning benefits when compared to single-time intervention models (9). The observed improvement in self-confidence for specific parameters is an important factor to consider, as this might lead encouraging practitioners to start utilizing hypofractionation. According to our current results, only ~11 – 15% of participants received prior web-based training on SRS/SBRT, which can be seen as an opportunity from an optimistic point of view, yet realistically shows an enormous area of improvement. Including innovative approaches based on online lecturing, interactive learning and artificial intelligence might indeed improve educational outcomes, equating access possibilities for LMIC (17, 18). Interestingly, participants from institutions where a pre-course debriefing (4 on-site and 2 on-line) took place had increased adherence to the course, in comparison to those who did not (42.3% vs. 20.8%). This might suggest that initial sensitization through introductory talks, and describing the points to develop during the curriculum and potential benefits, would improve attendance rates. In addition, encouraging a spirit of community learning, while building stronger scientific networks, enhancing the educational process (19).

The overall outcomes in terms of self-confidence and knowledge levels, for implementing or participating in SBRT/SRS programs, significantly improved throughout the development of the course. The importance of increasing confidence and knowledge amongst practitioners lies not only on their personal expectancies and capacities (20), but also on the impact on patient’s lives and clinical outcomes. By bridging accessibility to experts from other regions, a wider panorama opens for external knowledge and experience, enriching insights to improve treatment quality. A clear example of clinical influence could be taken from a previous study on longitudinal intensive-care education in developing countries. Results from this study demonstrated reduced post-intervention overall mortality (43% vs. 27%), in-hospital mortality (51% vs. 44%), hospital stay (8.3 vs 3.6 days) and increased monetary savings ($400,000/2 years) (21).

Remarkably, increased improvement was found for medical physicists, residents and radiation therapists on an individual competence level, possibly implying a path for further interventions with higher emphasis on these professional groups. This might also pose a critical area of interest, in terms of educational goals, suggesting that granting access to training tools for this specific sub-group requires increased attention. On the contrary, no major modifications were seen in the radiation oncologist group in terms of baseline practicing confidence levels and changes throughout the curriculum development. As reported in our previous study, participants with higher initial confidence levels appeared to benefit to a smaller degree than those with lower initial levels.

The pre- vs. post-curriculum exam analysis yielded noteworthy outcomes. Increasing and consolidating knowledge were proven feasible through telehealth methods. The significant increase in mean Likert scale results confirm the hypothesis first established during our pilot program. Previous studies in developing countries also support these outcomes (22, 23).

With regard to the educators, it is noteworthy that 13 out of 16 lecturers from our pilot SBRT/SRS study in this second phase program. All educators previously indicated high satisfaction, gratification, and 100% willingness to volunteer in further courses. One of the noted biggest satisfiers was the flexibility to arrange their usually busy schedules conveniently.

Initiatives like ours are currently being repeated globally, in different RT-related topics. Sustainability of such approaches must be ensured through cooperation between different groups, encompassing practitioners, industry members, and university-affiliated institutions. Adding efforts between these societies will enhance the potential reach and impact of these international courses, while granting access to a larger number of participants. The recently launched Global Coalition for Radiotherapy, joining forces with RCC, will ease the path towards expanding educational opportunities in radiation oncology worldwide (24). Furthermore, initiatives to standardize training in RT are currently undergoing, aiming to establish common baseline competencies for practitioners across the globe. Hence, the first Specialty Portfolio in Radiation Oncology, has been recently released, providing a roadmap logbook for both trainees and trainers (25). Incorporating this manual into RCC’s further educational developments will certainly influence the acquisition of competences and common language in RT for LMICs. Increasing skills of local leaders is equally relevant, as their mentorship insights are of great value to adjust the educational programs to local needs.

Certain drawbacks should also be mentioned. The data obtained for this investigation can be accurately reproduced in most of Latam-LMICs. However, it is possible that outcomes in the different regions are inconsistent because of variations in baseline educational levels or other barriers which need to be overcome prior to initiating training courses. A locational analysis should be performed according to the inherent necessities in different regions and training programs should be accounted for them. For instance, a known situation of high workloads in LMICs might impair regular attendance to telehealth courses. Coordination between organizers and institutions must be performed upfront, in order to guarantee educational time slots that minimally alter clinical performance.

There are a variety of different approaches to assess competence or improvement of knowledge following didactic learning. The method we chose here, Likert scale-based measurements, provides a quantitative tool for assessing the level of confidence and didactic knowledge gleaned focusing on pre- and post-training surveys. New strategies for measurable impact on clinical practice need to be implemented. For our pilot educational program, we chose to implement didactic lectures. Nevertheless, we recognize that didactic may be limited with regard to extrapolation of the knowledge to the clinical environment. Consequently, we are now working on other practical approaches to supplement the didactic learning, including virtual demonstrations of modern treatment planning methods, videos and on-line demonstrations of quality assurance measurements of treatment machines, overall techniques for quality control of the treatment process in radiation oncology, amongst others. Furthermore, cloud-based measurement tools, for both medical and technical spheres, are currently being implemented in cooperation with industry actors in order to longitudinally assess practice change patterns after educational interventions.

Despite the challenges associated with our pilot educational program, we are highly encouraged by the results and firmly believe that longitudinal telehealth educational programs such as ours will become an effective educational standard in developing countries in the future.

## Conclusion

Longitudinal telehealth lecturing is an effective method for improving confidence and knowledge on SBRT/SRS amongst RT-related professionals. Remote continuing medical education should be widely adopted in lower-middle income countries.

## Data Availability Statement

The original contributions presented in the study are included in the article/**Supplementary Material**. Further inquiries can be directed to the corresponding author.

## Author Contributions

GRS, data collection, statistical analysis and interpretation. Manuscript draft and editing writing. RT and MH, data interpretation, manuscript draft and editing. SM and BC, program organization, manuscript editing. RC, data collection statistical analysis. Manuscript draft and review. DM, data curation, project administration. GJS, investigation conceptualization, methodology, supervision, and validation of manuscript writing. FG, manuscript revision and edition. IC, concept design and study development, manuscript editing and drafting. DR, concept design and study development, manuscript editing. BL, investigation conceptualization, methodology and design, data collection and curation, supervision, validation and editing of manuscript. All authors contributed to the article and approved the submitted version.

## Conflict of Interest

GRS, grants and personal fees from Carl Zeiss Meditec AG and personal fees from Roche Pharma AG, not related to this work. GJS, personal fees from Carl Zeiss Meditec AG, not related to this work. FG reports research grants and travel expenses from ELEKTA AB, grants, stocks, travel expenses and honoraria from NOXXON Pharma AG, research grants, travel expenses and honoraria from Carl Zeiss Meditec AG, travel expenses and honoraria from Bristol-Myers Squibb, Roche Pharma AG, MSD Sharp and Dohme GmbH and AstraZeneca GmbH, non-financial support from Oncare GmbH and Opasca GmbH. IC, research grants and travel expenses from Varian Medical Systems, Inc., research grants from Phillips Healthcare. GRS, RT, MH, SM, BC, RC, DM, IC, DR, and BL are volunteer non-paid members of Rayos Contra Cancer, Inc.

The remaining authors declare that the research was conducted in the absence of any commercial or financial relationships that could be construed as a potential conflict of interest.

## Publisher’s Note

All claims expressed in this article are solely those of the authors and do not necessarily represent those of their affiliated organizations, or those of the publisher, the editors and the reviewers. Any product that may be evaluated in this article, or claim that may be made by its manufacturer, is not guaranteed or endorsed by the publisher.
