# Supplementary material for: Longitudinal Remote SBRT/SRS Training in Latin America: A Prospective Cohort Study
Source: Front Oncol. 2022 Apr 11;12:851849. doi: 10.3389/fonc.2022.851849 (PMC9035934; doi:10.3389/fonc.2022.851849)
Supplement: Supplementary file 1 [file DataSheet_1.zip › Appendix 1.PDF]

Rayos Contra Cancer
Project ECHO Training Curriculum
Hypofractionation Techniques: SBRT and SRS

Physics and Radiobiology

1. Radiobiology of high dose per fraction

https://www.youtube.com/watch?v=4-26Vwl83K4&list=PLO7opt4dQZ05qP5P7HRS-BlPwwQ5Lndmv&index=1

2. *Introduction to ProKnow*

https://drive.google.com/open?id=1oPBdsfKMD0S17tLWPhrhVVdyMgOK290R

3. Physics of small fields (Hypofractionation)
    a. Physics of high dose per fraction (SBRT/SRS)
    b. Measurements
    c. Technical factors (Detectors, correction factors, etc.)
    d. clinical applications (separate lecture perhaps)

https://www.youtube.com/watch?v=OtKrl7RJ9_Q&list=PLO7opt4dQZ05qP5P7HRS-BlPwwQ5Lndmv&index=2

Simulation

4. Intracranial and spine
    a. Patient immobilization and CT acquisition

https://www.youtube.com/watch?v=DJEYRSn7iug&list=PLO7opt4dQZ05qP5P7HRS-BlPwwQ5Lndmv&index=3

5. Extracranial and body sites
    a. (e.g. lung, liver, management and motion)
    Patient immobilization and CT acquisition

https://www.youtube.com/watch?v=503NCBOeUr8&list=PLO7opt4dQZ05qP5P7HRS-BlPwwQ5Lndmv&index=4

Case-Based Learning

6. Brain SRS

https://www.youtube.com/watch?v=NJJaAo7XAlo&list=PLO7opt4dQZ05qP5P7HRS-BlPwwQ5Lndmv&index=5

Dosimetry and Treatment Planning

7. Extracranial SBRT physics considerations
    a. Extracranial Clinical applications
    b. Treatment planning and dose calculation (motion management, single iso, multiple iso, IMRT, Rapid Arc, etc.)
    c. Plan evaluation
    d. Challenges

https://www.youtube.com/watch?v=aPP90AxBJbE&list=PLO7opt4dQZ05qP5P7HRS-BlPwwQ5Lndmv&index=6
(English version)
https://www.youtube.com/watch?v=nOcvVJ311lo&list=PLO7opt4dQZ05qP5P7HRS-BlPwwQ5Lndmv&index=7
(Spanish version)

8. Intracranial SRS physics considerations
    a. Intracranial Clinical applications
    b. Treatment planning and dose calculation (single iso, multiple iso, IMRT, Rapid Arc, etc.)
    c. Plan evaluation
    d. Challenges

https://www.youtube.com/watch?v=duFMnR7P4xg&list=PLO7opt4dQZ05qP5P7HRS-BlPwwQ5Lndmv&index=8

IGRT and Treatment Delivery

9. Intracranial and Spine
    a. immobilization (framed vs. frameless, etc.)
    b. delivery techniques (Gamma-Knife, Cyber-Knife, Linac-based)

c. setup uncertainties
d. image guided techniques
https://www.youtube.com/watch?v=IWA3ijUx5qk&list=PLO7opt4dQZ05qP5P7HRS-BlPwwQ5Lndmv&index=9

Case Based Learning
10. Clinical pearls of Hypofractionation
https://www.youtube.com/watch?v=QsKBc3PF6AA&list=PLO7opt4dQZ05qP5P7HRS-BlPwwQ5Lndmv&index=10

11. Extracranial and body (Part 1 – Lung)
a. immobilization
b. delivery techniques (Gamma-Knife, Cyber-Knife, Linac-based)
https://www.youtube.com/watch?v=UsDxOGsRXlY&list=PLO7opt4dQZ05qP5P7HRS-BlPwwQ5Lndmv&index=11

12. Extracranial and body (Part 2 – Liver and Pancreas)
b. setup uncertainties
c. image guided techniques
https://www.youtube.com/watch?v=9PToQb2eKco&list=PLO7opt4dQZ05qP5P7HRS-BlPwwQ5Lndmv&index=12

Case Based Learning
13. Prostate – (**Hiram Gay**)
https://www.youtube.com/watch?v=J6nrIKEDej8&list=PLO7opt4dQZ05qP5P7HRS-BlPwwQ5Lndmv&index=13

14. Question-based review and explanations
https://www.youtube.com/watch?v=BgeOKB6-Qk0&list=PLO7opt4dQZ05qP5P7HRS-BlPwwQ5Lndmv&index=14

Intra- and Extra-Cranial Quality Assurance
15. QA: Simulation, Treatment planning, Treatment Delivery, and Image Guidance
https://www.youtube.com/watch?v=oWgb9fQYpT0&list=PLO7opt4dQZ05qP5P7HRS-BlPwwQ5Lndmv&index=15

16. QA: IGRT
https://www.youtube.com/watch?v=3YNlED6w3Mk&list=PLO7opt4dQZ05qP5P7HRS-BlPwwQ5Lndmv&index=16

Case-Based Learning*
*Participating centers invited to upload and share their own cases. Allows for review of protocols, contours, planning considerations, and more during calls.
