# Supplementary material for: Longitudinal Remote SBRT/SRS Training in Latin America: A Prospective Cohort Study
Source: Front Oncol. 2022 Apr 11;12:851849. doi: 10.3389/fonc.2022.851849 (PMC9035934; doi:10.3389/fonc.2022.851849)
Supplement: Supplementary file 1 [file DataSheet_1.zip › Appendix 2.PDF]

SBRT/SRS 2.0 Pre-Curriculum Assessment

Welcome to the Rayos Contra Cancer SBRT/SRS 2.0 Curriculum. Please complete this form for enrollment.

Thank you!

---

1) Nombre ________________________________

2) Apellido ________________________________

3) Correo Electrónico ________________________________

4) Cual o cuales son sus roles actuales en el departamento? Marque todos que aplican.

- ☐ Jefe de física médica
- ☐ Físico médico
- ☐ Físico médico asistente
- ☐ Físico médico en entrenamiento
- ☐ en programa de Maestría
- ☐ en programa de PhD
- ☐ Profesor de física médica
- ☐ Dosimetrista
- ☐ Tecnólogo médico
- ☐ Residente
- ☐ Radioncologo
- ☐ Otro

5) Si otra, explique: ________________________________

6) Institución: ________________________________

7) Tipos de entrenamiento en radioterapia estereotáctica del cuerpo (SBRT). (Marque todas las que apliquen)

- ☐ No tiene entrenamiento
- ☐ Módulos en línea (via internet)
- ☐ Estudio independiente
- ☐ Talleres o entrenamiento en conferencias
- ☐ Entrenamiento formal por la industria o fabricante de equipos
- ☐ Académico formal

8) Tipos de entrenamiento en radiocirugía estereotáctica (SRS). (Marque todas las que apliquen)

- ☐ No tiene entrenamiento
- ☐ Módulos en línea (via internet)
- ☐ Estudio independiente
- ☐ Talleres o entrenamiento en conferencias
- ☐ Entrenamiento formal por la industria o fabricante de equipos
- ☐ Académico formal

9) Yo tengo confianza para desarrollar casos de SBRT.

- ○ Desacuerdo total
- ○ En desacuerdo
- ○ Neutral
- ○ En acuerdo
- ○ Acuerdo total

10) Yo tengo confianza para desarrollar casos de SRS.
- ◯ Desacuerdo total
- ◯ En desacuerdo
- ◯ Neutral
- ◯ En acuerdo
- ◯ Acuerdo total

11) ¿Cómo describiría su conocimiento actual para ejecutar un programa SBRT?
- ◯ Sin conocimiento
- ◯ Conocimiento mínimo
- ◯ Conocimiento promedio
- ◯ Conocimiento significativo
- ◯ Conocimiento experto

12) ¿Cómo describiría su conocimiento actual para ejecutar un programa SRS?
- ◯ Sin conocimiento
- ◯ Conocimiento mínimo
- ◯ Conocimiento promedio
- ◯ Conocimiento significativo
- ◯ Conocimiento experto

13) ¿Cómo describiría su capacidad actual para enseñar a otro físico cómo ser seguro en la SBRT?
- ◯ Sin habilidad
- ◯ Habilidad mínima
- ◯ Habilidad media
- ◯ Habilidad significativa
- ◯ Habilidad experta

14) ¿Cómo describiría su capacidad actual para enseñar a otro físico cómo ser seguro en la SRS?
- ◯ Sin habilidad
- ◯ Habilidad mínima
- ◯ Habilidad media
- ◯ Habilidad significativa
- ◯ Habilidad experta

Describa su nivel actual de confianza con los siguientes temas en una escala de 1-5 (1 = sin confianza, 2 = un poco de confianza, 3 = confianza moderada, 4 = muy confianza y 5 = confianza extrema):

	1	2	3	4	5
15) Implementar un programa SBRT desde el principio hasta el final	◯	◯	◯	◯	◯
16) Entender la física de la SBRT	◯	◯	◯	◯	◯
17) Entender el proceso de simulación y control del movimiento de SBRT	◯	◯	◯	◯	◯
18) Revisar la dosimetria del tratamiento de SBRT	◯	◯	◯	◯	◯
19) Planificación del tratamiento de SBRT	◯	◯	◯	◯	◯
20) Entender la utilidad de IGRT para SBRT	◯	◯	◯	◯	◯
21) Entender los requisitos de infraestructura, hardware y software	◯	◯	◯	◯	◯

22)

	Entender el comisionamiento de SBRT	○	○	○	○	○
23)	Entender cómo se realiza la garantía de calidad para la SBRT	○	○	○	○	○
24)	Entender las aplicaciones clínicas (relacionadas con pacientes y enfermedades) de SBRT	○	○	○	○	○

---

25) Por favor mencione cualquier comentario adicional que desee compartir con nosotros.

____

SBRT/SRS 2.0 Pre-Curriculum Assessment

Welcome to the Rayos Contra Cancer SBRT/SRS 2.0 Curriculum. Please complete this form for enrollment.

Thank you!

---

1) First Name ______________________

2) Last Name ______________________

3) Email Address ______________________

4) What is (are) your current role(s) in the department? Check all that apply.
- ☐ Chief medical physicist
- ☐ Senior medical physicist
- ☐ Junior medical physicist
- ☐ Medical physicist in training
- ☐ Master's Program
- ☐ PhD Program
- ☐ Medical Physics Professor
- ☐ Dosimetrist
- ☐ Radiation Therapist
- ☐ Resident
- ☐ Radiation Oncologist
- ☐ Other

5) If other, please explain: ______________________

6) Institution: ______________________

7) Types of training in stereotactic body radiotherapy (SBRT). (Check all that apply)
- ☐ No training
- ☐ Online modules
- ☐ Independent study
- ☐ Conference workshop or training
- ☐ Formal training from industry or equipment manufacturer
- ☐ Formal academic setting

8) Types of training in stereotactic radiosurgery (SRS). (Check all that apply)
- ☐ No training
- ☐ Online modules
- ☐ Independent study
- ☐ Conference workshop or training
- ☐ Formal training from industry or equipment manufacturer
- ☐ Formal academic setting

9) I have confidence in developing SBRT cases.
- ○ Strongly disagree
- ○ Disagree
- ○ Neither agree nor disagree
- ○ Agree
- ○ Strongly agree

10) I have confidence developing SRS cases.
- ◯ Strongly disagree
- ◯ Disagree
- ◯ Neither agree nor disagree
- ◯ Agree
- ◯ Strongly agree

11) How would you describe your knowledge in executing an SBRT program?
- ◯ No knowledge
- ◯ Minimal knowledge
- ◯ Sufficient knowledge
- ◯ Significant knowledge
- ◯ Expert knowledge

12) How would you describe your knowledge in executing an SRS program?
- ◯ No knowledge
- ◯ Minimal knowledge
- ◯ Sufficient knowledge
- ◯ Significant knowledge
- ◯ Expert knowledge

13) How would you describe your ability to teach another physicist how to ensure safety in SBRT?
- ◯ No ability
- ◯ Minimal ability
- ◯ Medium ability
- ◯ Significant ability
- ◯ Expert ability

14) How would you describe your ability to teach another physicist how to ensure safety in SRS?
- ◯ No ability
- ◯ Minimal ability
- ◯ Medium ability
- ◯ Significant ability
- ◯ Expert ability

Describe your level of confidence with the following subjects on a scale of 1-5 (1= no confidence, 2= slightly confident, 3= moderately confident, 4= very confident, 5= extremely confident).

	1	2	3	4	5
15) Implement an SBRT program from beginning to end	◯	◯	◯	◯	◯
16) Understand the physics of SBRT	◯	◯	◯	◯	◯
17) Understand the simulation and immobilization process of SBRT	◯	◯	◯	◯	◯
18) Review the dosimetry of SBRT treatment	◯	◯	◯	◯	◯
19) SBRT treatment planning	◯	◯	◯	◯	◯
20) Understand the role of IGRT for SBRT	◯	◯	◯	◯	◯
21) Understand the requirements for infrastructure, hardware, and software	◯	◯	◯	◯	◯
22) Understand the commissioning of SBRT	◯	◯	◯	◯	◯

23)

	Understand how quality assurance is performed for SBRT	○	○	○	○	○
24)	Understand the clinical applications (related to patients and diseases) of SBRT	○	○	○	○	○

---

25) Please provide any additional comments you would like to share with us.

____

SBRT/SRS 2.0 Post-Curriculum Assessment

Thank you for participating in the SBRT/SRS 2.0 Curriculum. Please fill out this form to complete enrollment.

Thank you!

1) Nombre ______________________________

2) Apellido ______________________________

3) Correo Electrónico ______________________________

4) Cual o cuales son sus roles actuales en el departamento? Marque todos que aplican.

- ☐ Jefe de física médica
- ☐ Físico médico
- ☐ Físico médico asistente
- ☐ Físico médico en entrenamiento
- ☐ en programa de Maestría
- ☐ en programa de PhD
- ☐ Profesor de física médica
- ☐ Dosimetrista
- ☐ Tecnólogo médico
- ☐ Residente
- ☐ Radioncologo
- ☐ Otro

5) Si otra, explique: ______________________________

6) Institución: ______________________________

7) Tipos de entrenamiento en radioterapia estereotáctica del cuerpo (SBRT). (Marque todas las que apliquen)

- ☐ No tiene entrenamiento
- ☐ Módulos en línea (via internet)
- ☐ Estudio independiente
- ☐ Talleres o entrenamiento en conferencias
- ☐ Entrenamiento formal por la industria o fabricante de equipos
- ☐ Académico formal

8) Tipos de entrenamiento en radiocirugía estereotáctica (SRS). (Marque todas las que apliquen)

- ☐ No tiene entrenamiento
- ☐ Módulos en línea (via internet)
- ☐ Estudio independiente
- ☐ Talleres o entrenamiento en conferencias
- ☐ Entrenamiento formal por la industria o fabricante de equipos
- ☐ Académico formal

9) Yo tengo confianza para desarrollar casos de SBRT.

- ◯ Desacuerdo total
- ◯ En desacuerdo
- ◯ Neutral
- ◯ En acuerdo
- ◯ Acuerdo total

10) Yo tengo confianza para desarrollar casos de SRS.
- ◯ Desacuerdo total
- ◯ En desacuerdo
- ◯ Neutral
- ◯ En acuerdo
- ◯ Acuerdo total

11) ¿Cómo describiría su conocimiento actual para ejecutar un programa SBRT?
- ◯ Sin conocimiento
- ◯ Conocimiento mínimo
- ◯ Conocimiento promedio
- ◯ Conocimiento significativo
- ◯ Conocimiento experto

12) ¿Cómo describiría su conocimiento actual para ejecutar un programa SRS?
- ◯ Sin conocimiento
- ◯ Conocimiento mínimo
- ◯ Conocimiento promedio
- ◯ Conocimiento significativo
- ◯ Conocimiento experto

13) ¿Cómo describiría su capacidad actual para enseñar a otro físico cómo ser seguro en la SBRT?
- ◯ Sin habilidad
- ◯ Habilidad mínima
- ◯ Habilidad media
- ◯ Habilidad significativa
- ◯ Habilidad experta

14) ¿Cómo describiría su capacidad actual para enseñar a otro físico cómo ser seguro en la SRS?
- ◯ Sin habilidad
- ◯ Habilidad mínima
- ◯ Habilidad media
- ◯ Habilidad significativa
- ◯ Habilidad experta

Describa su nivel actual de confianza con los siguientes temas en una escala de 1-5 (1 = sin confianza, 2 = un poco de confianza, 3 = confianza moderada, 4 = muy confianza y 5 = confianza extrema):

	1	2	3	4	5
15) Implementar un programa SBRT desde el principio hasta el final	◯	◯	◯	◯	◯
16) Entender la física de la SBRT	◯	◯	◯	◯	◯
17) Entender el proceso de simulación y control del movimiento de SBRT	◯	◯	◯	◯	◯
18) Revisar la dosimetria del tratamiento de SBRT	◯	◯	◯	◯	◯
19) Planificación del tratamiento de SBRT	◯	◯	◯	◯	◯
20) Entender la utilidad de IGRT para SBRT	◯	◯	◯	◯	◯
21) Entender los requisitos de infraestructura, hardware y software	◯	◯	◯	◯	◯

22)

	Entender el comisionamiento de SBRT	◯	◯	◯	◯	◯
23)	Entender cómo se realiza la garantía de calidad para la SBRT	◯	◯	◯	◯	◯
24)	Entender las aplicaciones clínicas (relacionadas con pacientes y enfermedades) de SBRT	◯	◯	◯	◯	◯

25) ¿Qué es una cosa que usted aprendió durante el SBRT/SRS 2.0 currículo que es especialmente memorable?

____

26) Si corresponde, ¿cuál es un cambio específico que su clínica ha implementado como resultado del SBRT/SRS 2.0 currículo? Describa uno de los cambios que cree que tendrán un impacto positivo en la atención al paciente.

____

27) ¿Qué aspecto del currículo ha sido el más útil? Describa uno o más aspectos que han sido útiles.

____

28) ¿Tiene algún feedback o otros comentarios?

____

English - SBRT/SRS 2.0 Post-Curriculum Assessment

Welcome to the Rayos Contra Cancer SBRT/SRS 2.0 Curriculum. Please complete this form for enrollment.

Thank you!

---

1) First Name

____

---

2) Last Name

____

---

3) Email Address

____

---

4) What is (are) your current role(s) in the department? Check all that apply.

- ☐ Chief medical physicist
- ☐ Senior medical physicist
- ☐ Junior medical physicist
- ☐ Medical physicist in training
- ☐ Master's Program
- ☐ PhD Program
- ☐ Medical Physics Professor
- ☐ Dosimetrist
- ☐ Radiation Therapist
- ☐ Resident
- ☐ Radiation Oncologist
- ☐ Other

---

5) If other, please explain:

____

---

6) Institution:

____

---

7) Types of training in stereotactic body radiotherapy (SBRT). (Check all that apply)

- ☐ No training
- ☐ Online modules
- ☐ Independent study
- ☐ Conference workshop or training
- ☐ Formal training from industry or equipment manufacturer
- ☐ Formal academic setting

---

8) Types of training in stereotactic radiosurgery (SRS). (Check all that apply)

- ☐ No training
- ☐ Online modules
- ☐ Independent study
- ☐ Conference workshop or training
- ☐ Formal training from industry or equipment manufacturer
- ☐ Formal academic setting

---

9) I have confidence in developing SBRT cases.

- ◯ Strongly disagree
- ◯ Disagree
- ◯ Neither agree nor disagree
- ◯ Agree
- ◯ Strongly agree

10) I have confidence developing SRS cases.
- ◯ Strongly disagree
- ◯ Disagree
- ◯ Neither agree nor disagree
- ◯ Agree
- ◯ Strongly agree

11) How would you describe your knowledge in executing an SBRT program?
- ◯ No knowledge
- ◯ Minimal knowledge
- ◯ Sufficient knowledge
- ◯ Significant knowledge
- ◯ Expert knowledge

12) How would you describe your knowledge in executing an SRS program?
- ◯ No knowledge
- ◯ Minimal knowledge
- ◯ Sufficient knowledge
- ◯ Significant knowledge
- ◯ Expert knowledge

13) How would you describe your ability to teach another physicist how to ensure safety in SBRT?
- ◯ No ability
- ◯ Minimal ability
- ◯ Medium ability
- ◯ Significant ability
- ◯ Expert ability

14) How would you describe your ability to teach another physicist how to ensure safety in SRS?
- ◯ No ability
- ◯ Minimal ability
- ◯ Medium ability
- ◯ Significant ability
- ◯ Expert ability

Describe your level of confidence with the following subjects on a scale of 1-5 (1= no confidence, 2= slightly confident, 3= moderately confident, 4= very confident, 5= extremely confident).

	1	2	3	4	5
15) Implement an SBRT program from beginning to end	◯	◯	◯	◯	◯
16) Understand the physics of SBRT	◯	◯	◯	◯	◯
17) Understand the simulation and immobilization process of SBRT	◯	◯	◯	◯	◯
18) Review the dosimetry of SBRT treatment	◯	◯	◯	◯	◯
19) SBRT treatment planning	◯	◯	◯	◯	◯
20) Understand the role of IGRT for SBRT	◯	◯	◯	◯	◯
21) Understand the requirements for infrastructure, hardware, and software	◯	◯	◯	◯	◯
22) Understand the commissioning of SBRT	◯	◯	◯	◯	◯

23)

	Understand how quality assurance is performed for SBRT	○	○	○	○	○
24)	Understand the clinical applications (related to patients and diseases) of SBRT	○	○	○	○	○

25) What is one thing you learned from the curriculum that is especially memorable?

____

26) If applicable, what is one specific change your clinic has implemented as a result of this curriculum? Please describe one of more changes that you feel will have positive impact on patient care.

____

27) What aspect of the curriculum has been the most helpful? Please describe one or more aspects that have been the helpful.

____

28) Do you have any feedback or other comments?

____

RCC SBRT/SRS 2.0 Pre-Curriculum Exam

* Required

1. Email address *

2. Name/Nombre Completo *

3. Institution/Institución *

4. Considering the "Rs" of radiobiology, choose the one CORRECT response.

   Mark only one oval.

   - Reassortment of cells in the cell cycle can explain the response of cancer cells to radiation
   - Repopulation of cells can explain the response of cancer cells to radiation exposures typical of hypofractionation.
   - Reoxygenation can explain the response of cancer cells to a single high dose of radiation.
   - Repair of DNA damage between fractions can explain, at least in part, the response of cancer cells to multiple hypofractionated doses of radiation.

5. Choose the one INCORRECT hypothesis put forth to explain how SRS can be so effective considering that 1) tumor hypoxia is a problem limiting the effectiveness of radiotherapy and 2) some metastatic brain tumors such as those arising from radioresistant primary cancers (melanoma, renal cell carcinoma, anaplastic thyroid cancer, etc.), respond as well to radiotherapy as radiosensitive cancers.

Mark only one oval.

- Tumor vessels are more sensitive to high dose radiation than low dose radiation
- Chronic anoxia occurs days after high dose radiation
- Immunological priming elicits an immune reaction after high dose radiation
- Reoxygenation is rapid and complete after high dose radiation

6. For Cone Based or Gamma knife based SRS treatments a crucial step in ensuring accuracy of targeting/delineating the tumor area is

Mark only one oval.

- MRI must be acquired before the CT
- CT scan must be acquired before MRI
- Assuring the Planning Framed CT scan and MRI are dimensionally correct and CO-registered appropriately.
- Ensuring the image resolution is the lowest in each imaging modality.

7. The key to conformality of dose around target is

Mark only one oval.

- Number of non-coplanar and non-opposing beams or beamlets
- MLC penumbras
- Proper use of PTV margins
- Decreasing the MLC Leaf width

8. For Single fraction SBRT –spine ; immobilization of the patient should be performed such that

Mark only one oval.

- The calculation grid of the planning CT scan can be reduced to less than 1mm
- Minimum INTRA fraction motion is expected and minimum INTRA fraction online imaging is necessary.
- No imaging of the patient is required for setup
- the co- registration of planning CT and treatment imaging is appropriate

9. Use of the wrong volume size detector for small field relative and/or absolute dosimetry will lead to possible?

Mark only one oval.

- Underestimate the absolute dose and underestimate the coverage of targets. This in turns translates to a possible overdosing of the patient.
- Overestimate the absolute dose and overestimate the coverage of targets. This in turns translates to a possible under dosing of the patient.
- Overestimate the absolute dose and underestimate the coverage of targets. This in turns translates to a possible overdosing of the patient.
- Underestimate the absolute dose and overestimate the coverage of targets. This in turns translates to a possible under dosing of the patient.

10. For IMRT SBRT-Lung minimizing the interplay effect in between target motion and radiation delivery can be BEST and Easiest accomplished by

Mark only one oval.

- Minimizing the target motion using immobilization techniques at the time of simulation.
- Using gating techniques during the exhale phase of the breathing cycle.
- Adding more margins for tumors than move more than 1 cm.
- Using appropriate external and internal surrogate marker.

11. Which of the following diseases can be treated with stereotactic radiosurgery?

Mark only one oval.

- a brain metastatic tumor of 1 cubic centimeters.
- an arterio-venous malformation of 25 cubic centimeters.
- a brain tumor volume of 100 cubic centimeters.
- a brain surgical cavity of 8 cubic centimeters.

12. Which of the following is not an imaging modality for use in intracranial radiosurgery simulation?

Mark only one oval.

- Ultrasound.
- Computed Tomography.
- Magnetic Resonance.
- Angiography.

13. Which of the following equipment is not used during stereotactic radiosurgery simulation?

Mark only one oval.

- ( ) Rigid and soft frames.
- ( ) Stereotactic phantoms.
- ( ) Stereotactic localizers.
- ( ) Target localization software.

14. Which of the following sentence is not true regarding stereotactic radiosurgery systems?

Mark only one oval.

- ( ) The GammaKnife system provides a robotic couch capable of correcting patient positioning in six dimensions (x,y,z and rotation: pitch, roll and yaw).
- ( ) Cyberknife uses non-invasive image guided target localization.
- ( ) Linear accelerators can use high-definition MLCs or cones for treatment.
- ( ) Dynamic conformal arcs can be used in linear accelerators for treatment.

15. Which of the following statements is true at field sizes less than 1x1 cm2:

Mark only one oval.

- ( ) lateral photon scattering decreases
- ( ) lateral electron scattering decreases
- ( ) beam energy energy decreases
- ( ) effective photon source becomes occluded

16. Which of the following statements regarding depth dose measurements for a 2x2 cm2, 6 MV field, in a water (4 cm)/lung (6 cm)/water (10 cm) phantom is True?

Mark only one oval.

- The dose in the lung slab will increase because the density of the lung tissue is lower than that of water.
- The dose in the lung slab will increase because of increased photon scattering in the lung tissue.
- The dose in the lung slab will decrease because the range of scattered photons increases in the lung tissue.
- The dose in the lung slab will decrease because the range of scattered electrons increases in the lung tissue.

17. Which of the following statements concerning image-guided treatment delivery is false

Mark only one oval.

- The treatment time for radiation delivery of a gated treatment is generally higher than that of a free-breathing treatment.
- Surface-based image guidance may sometimes be a poor surrogate for tumor motion.
- Patient alignment using volumetric imaging may help reduce treatment margins.
- Daily imaging using planar x-rays gives excellent information on the motion of the tumor and normal tissues.
- It is important that the strength of the imaging surrogate be considered in the planning margin design.

18. In CT-frame-based radiosurgery, what is the largest source of uncertainty?

Mark only one oval.

- Stereotactic frame setup
- Linac radiation isocenter accuracy
- CT image resolution
- Tissue motion
- Dose calculation algorithm

19. In a single-isocenter, conformal radiosurgery treatment of a symmetrical target, the dose gradient outside the target is sharpest for the prescription isodose line of:

Mark only one oval.

- 50%
- 70%
- 80%
- 90%
- 100%

20. Which of the following is an appropriate setup verification technique for a single fraction linac radiosurgery treatment?

Mark only one oval.

- Skin marks and tattoos
- Orthogonal MV images
- CBCT setup with 3D image registration to the planning CT
- Surface optical imaging
- Depth helmet measurements

21. According with the SRS ASTRO recommendations, what is the tolerance of a Winston Lutz test for SRS/SBRT?

Mark only one oval.

- 1.5 mm maximum
- 1.0 mm average
- 0.75 mm maximum
- 0.75 mm average
- 0.5 mm average

22. According with the SRS ASTRO recommendations, what's the frequency and tolerance for the IGRT positioning/repositioning?

Mark only one oval.

- Annually, ≤0.75 mm
- Monthly, ≤1.5 mm
- Monthly, ≤1.0 mm
- Daily, ≤1.5 mm
- Daily, ≤1.0 mm

23. Which of the following is NOT a method to reduce internal target motion in abdominal SBRT?

Mark only one oval.

- Breath-hold treatment
- Abdominal compression
- Increased margins
- Dynamic gating

24. Accurate target localization at time of treatment is best achieved through which pre-treatment imaging technique?

Mark only one oval.

- kV-kV pair
- 3D imaging (CT-on-rails or CBCT)
- Alignment to patient marks/tattoos
- MV port films

25. Which one is the gold standard tumor tracking in Radiotherapy?

Mark only one oval.

- Fiducials
- Calcification
- Beacons
- Tattoos

26. Which tumor tracking method is used by Cyberknife for intracranial lesions?

Mark only one oval.

- 6d Skull
- Point and shoot
- Fiducials
- Synchrony

27. For the daily output (cGy/MU) constancy check, what's the expected deviation from baseline?

Mark only one oval.

- ~ ± 3 %
- ~ ± 2 %
- ~ ± 1 %
- ~ ± 0.5 %

28. For the monthly output (cGy/MU) constancy check, what's the expected deviation from baseline?

Mark only one oval.

- ~ ± 3 %
- ~ ± 2 %
- ~ ± 1 %
- ~ ± 0.5 %

29. The robotic arm positioning error determined from film measurements should be approximately?

Mark only one oval.

- 2 mm
- 1.5 mm
- 1.0 mm
- 0.5 mm

30. Delivery of a Cyberknife treatment involves imaging the patient:

Mark only one oval.

- Prior to each field irradiation
- Via a CBCT before, during, and after treatment
- Via optical surface monitoring system (OSMS)
- None of the above

31. Standard clinical mask may be permissible with appropriate institutional margins and image-guidance. Uncertainties with masks, even specialized, can be:

Mark only one oval.

- about 0.3 mm
- up to 0.5 mm
- up to 1.0 mm
- up to 2 mm or more

32. “Highly Reliable Organizations,” have several key traits: sensitivity to operations, reluctancy to accept “simple” explanations for errors, preoccupation with failure, deference to expertise, and resiliency. Which is not an example of such a trait:

Mark only one oval.

- An efficient operational process is a safe process; straightforward and unambiguous quality and safety system with well-defined control points.
- Focus on failure in work process and culture, the primary factors for quality and safety.
- Normalization of Deviance, the slow acceptance of shortcuts in established safety processes.
- Use of the Deming cycle, “Plan-Do-Check-Act.” Use of a “loop process” to ensure continuous improvement and learning from errors and near-misses.

33. Stereotactic treatments require end-to-end tests on a regular basis, ideally embedded within a well-implemented PDCA (Plan-Do-Check-Act) continuous quality improvement cycle. An end-to-end test is best because:

Mark only one oval.

- It is difficult to determine the influence of an individual uncertainty; composite effects due to the total uncertainty are more relevant clinically.
- Establishing the total dosimetric and geometric uncertainty for an institution is useful for determining institutional margins and/or prescription isodose lines for treatments.
- Accuracy in absolute dose, dose distribution, patient geometry and target positioning can be verified within institutional limits.
- All of the above are reasons why the end-to-end test is a gold standard quality assurance test.

This content is neither created nor endorsed by Google.

Google Forms
