# Supplementary material for: Longitudinal Remote SBRT/SRS Training in Latin America: A Prospective Cohort Study
Source: Front Oncol. 2022 Apr 11;12:851849. doi: 10.3389/fonc.2022.851849 (PMC9035934; doi:10.3389/fonc.2022.851849)

Pre- (left) and post-curriculum (right) evaluations

Considering the "Rs" of radiobiology, choose the one CORRECT response.

88 von 141 richtigen Antworten

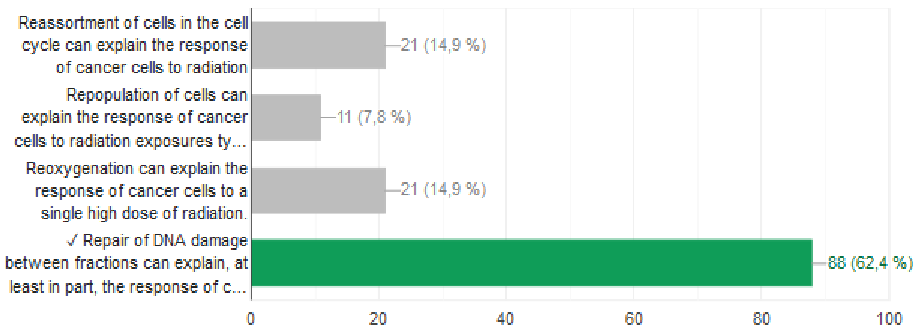

Considering the "Rs" of radiobiology, choose the one CORRECT response.

58 von 81 richtigen Antworten

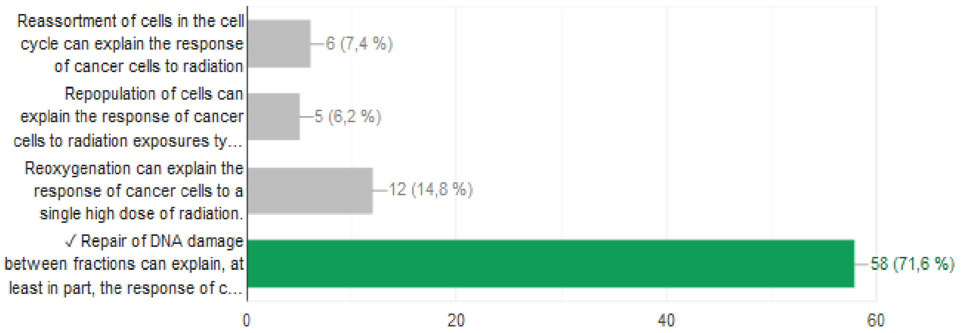

Choose the one INCORRECT hypothesis put forth to explain how SRS can be so effective considering that 1) tumor hypoxia is a problem limiting the effectiveness of radiotherapy and 2) some metastatic brain tumors such as those arising from radioresistant primary cancers (melanoma, renal cell carcinoma, anaplastic thyroid cancer, etc.), respond as well to radiotherapy as radiosensitive cancers.

84 von 140 richtigen Antworten

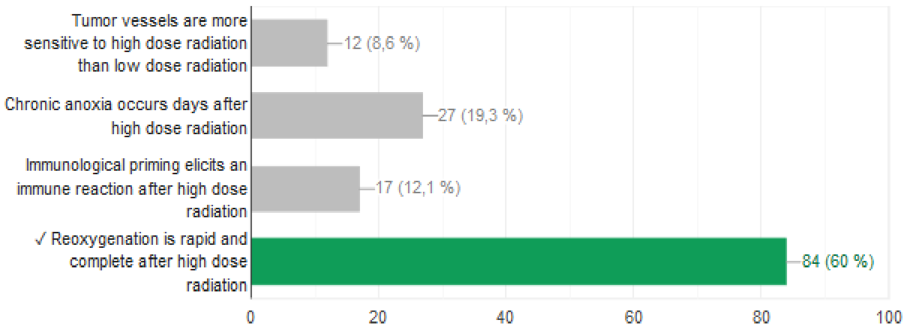

Choose the one INCORRECT hypothesis put forth to explain how SRS can be so effective considering that 1) tumor hypoxia is a problem limiting the effectiveness of radiotherapy and 2) some metastatic brain tumors such as those arising from radioresistant primary cancers (melanoma, renal cell carcinoma, anaplastic thyroid cancer, etc.), respond as well to radiotherapy as radiosensitive cancers.

65 von 81 richtigen Antworten

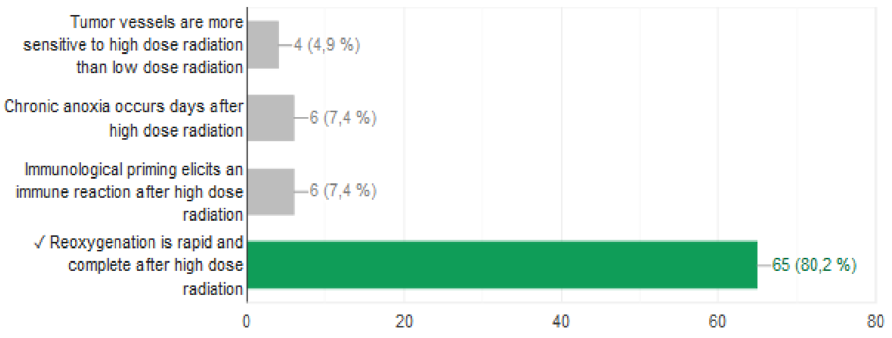

Pre- (left) and post-curriculum (right) evaluations

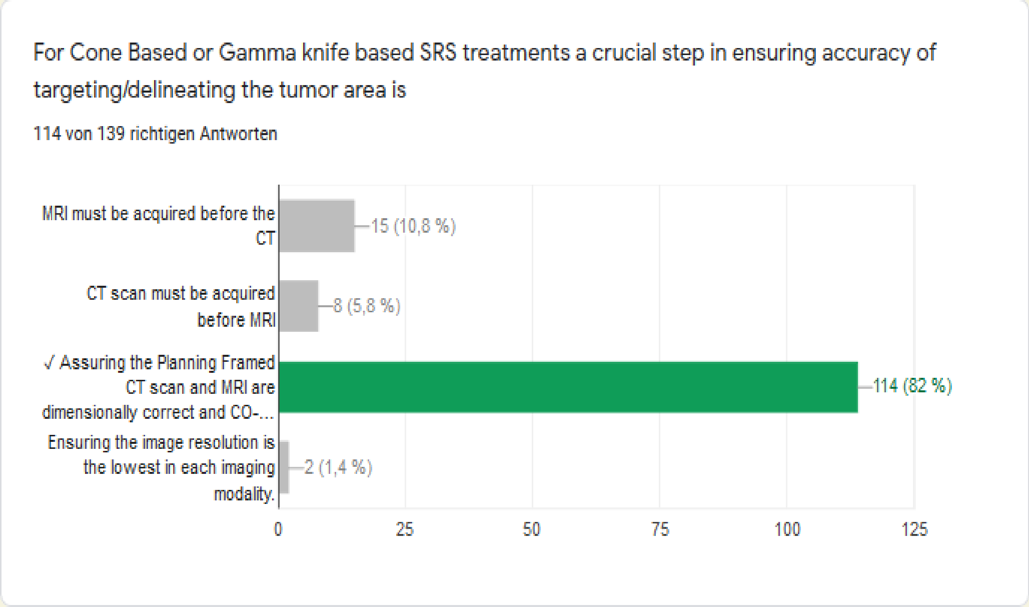

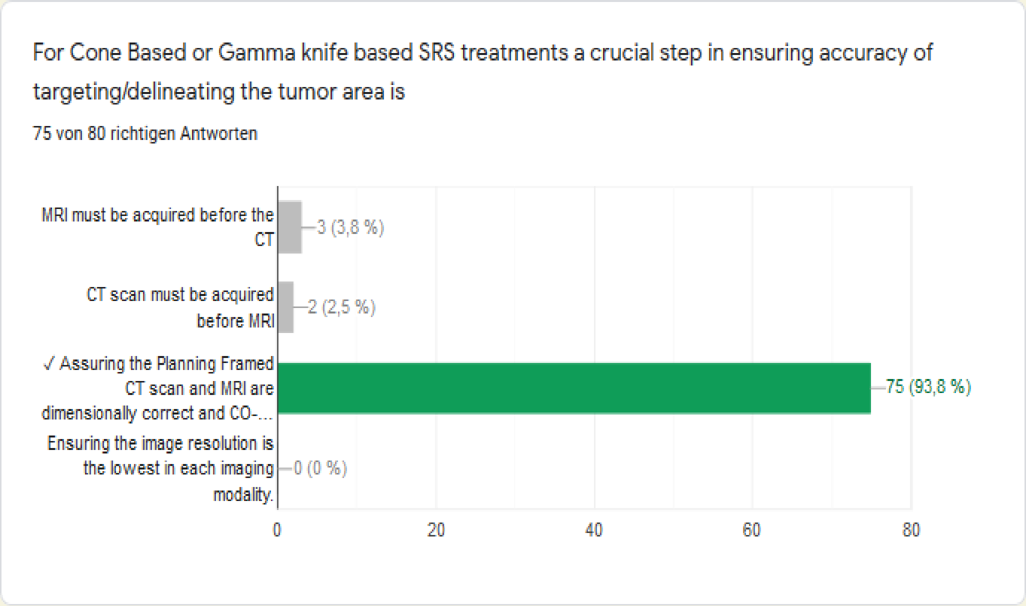

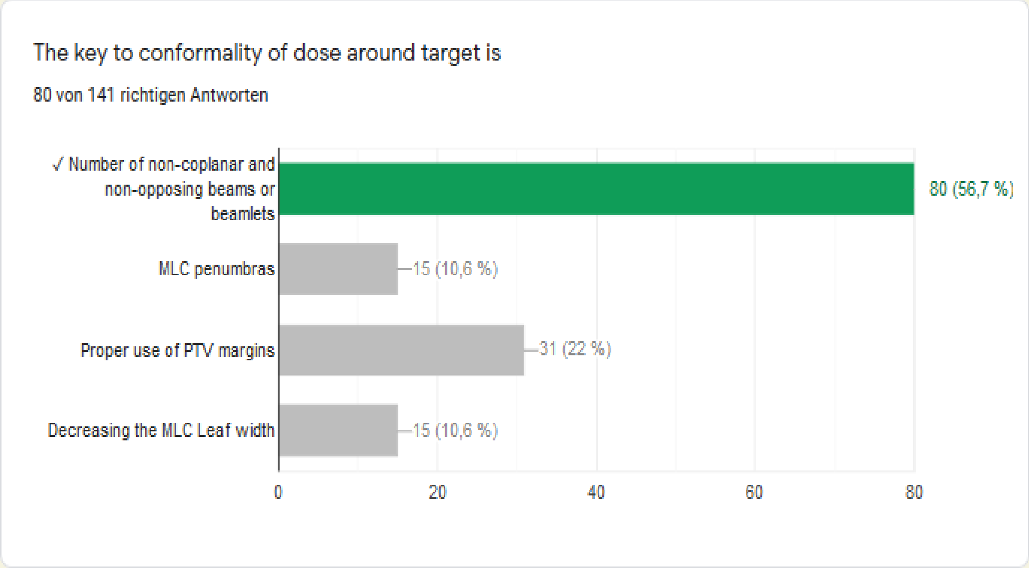

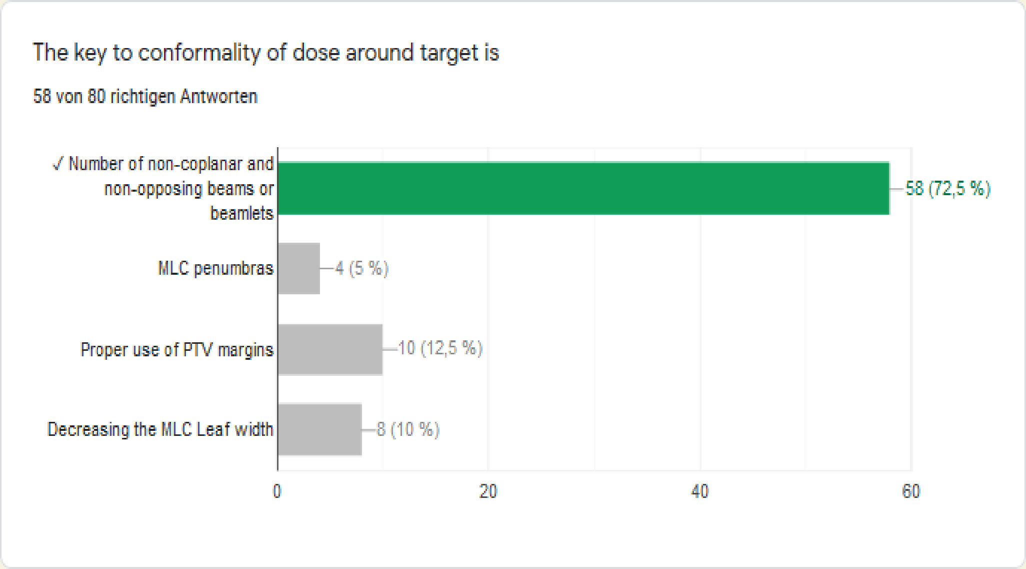

Pre- (left) and post-curriculum (right) evaluations

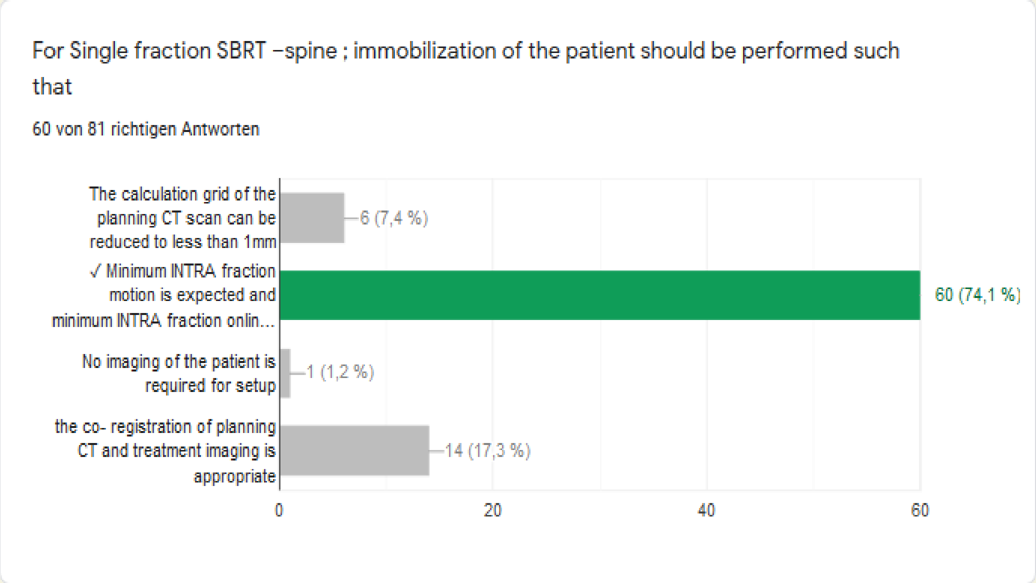

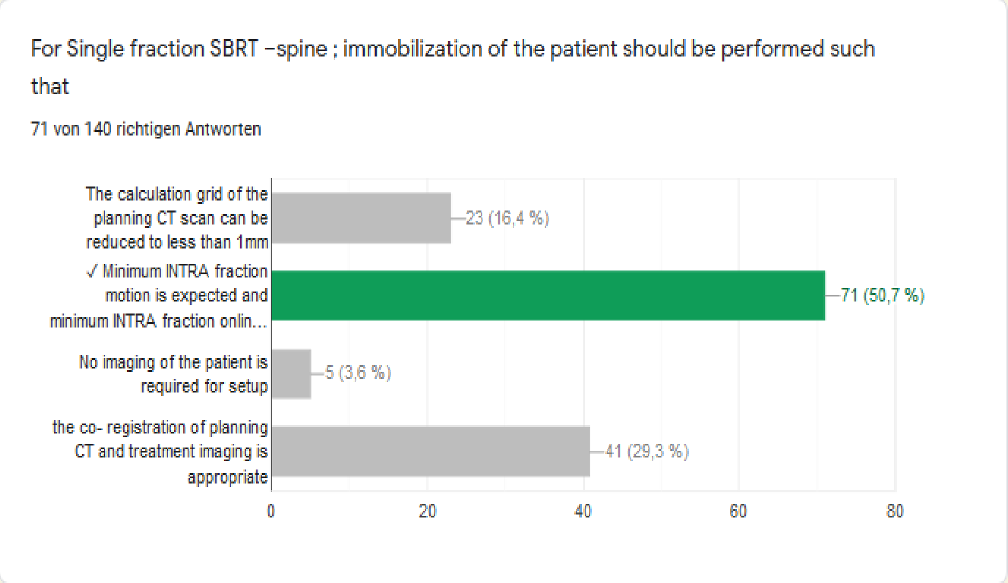

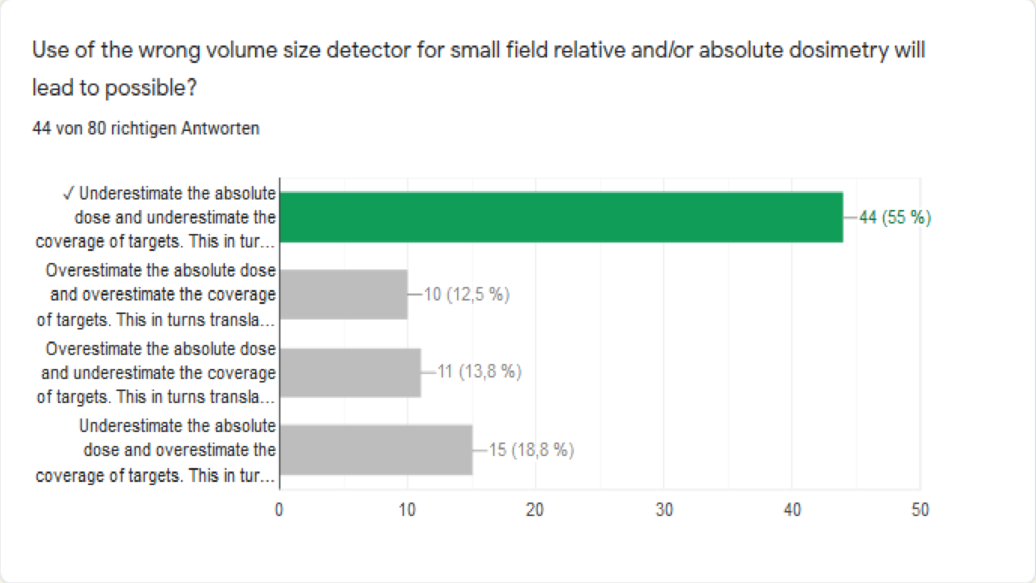

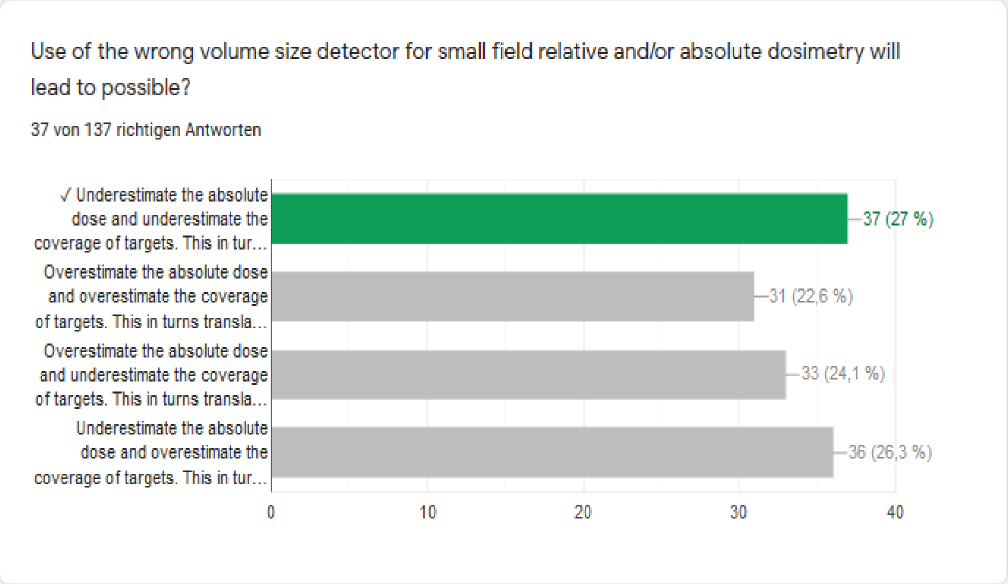

Pre- (left) and post-curriculum (right) evaluations

For IMRT SBRT-Lung minimizing the interplay effect in between target motion and radiation delivery can be BEST and Easiest accomplished by

60 von 138 richtigen Antworten

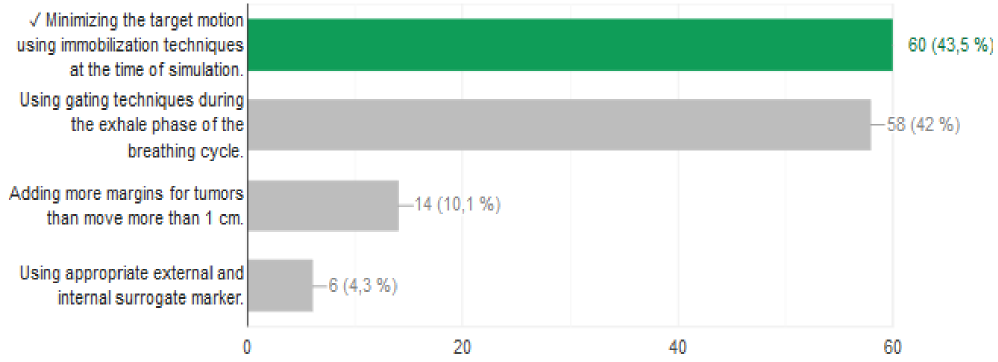

For IMRT SBRT-Lung minimizing the interplay effect in between target motion and radiation delivery can be BEST and Easiest accomplished by

57 von 80 richtigen Antworten

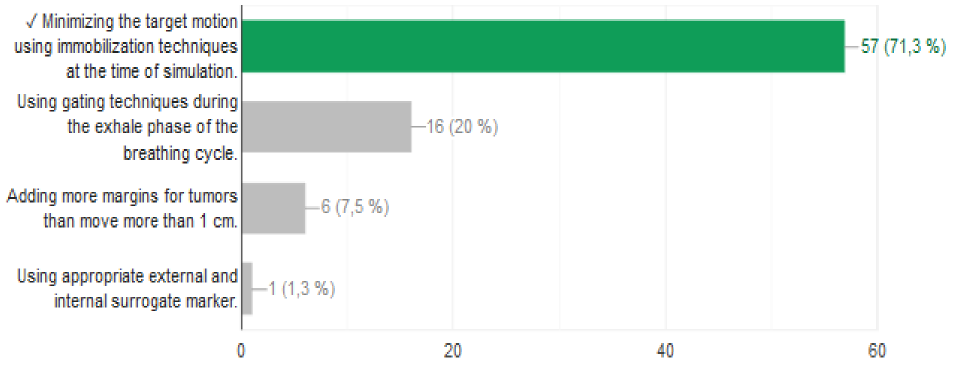

Which of the following diseases can be treated with stereotactic radiosurgery?

14 von 139 richtigen Antworten

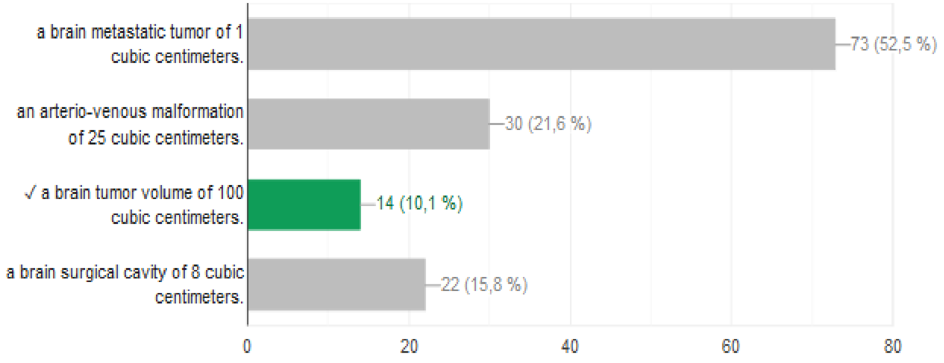

Which of the following diseases can not be treated with stereotactic radiosurgery?

63 von 81 richtigen Antworten

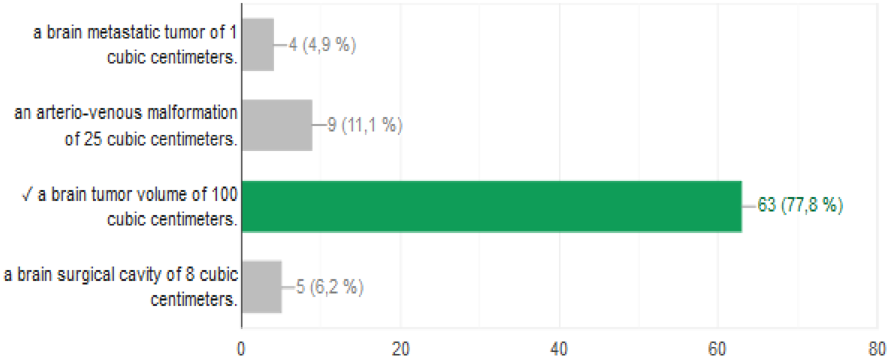

Pre- (left) and post-curriculum (right) evaluations

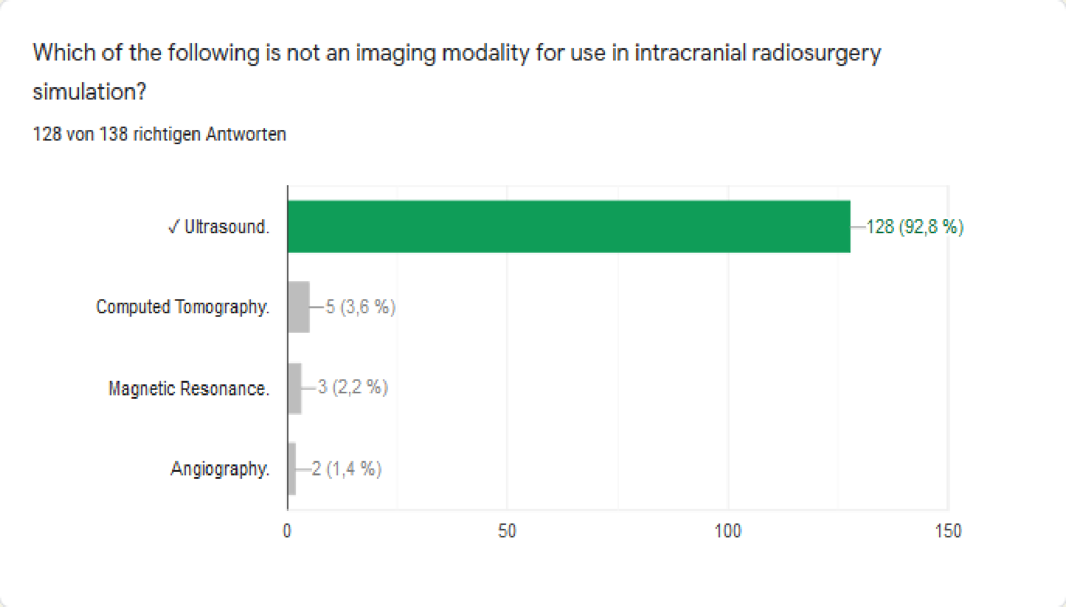

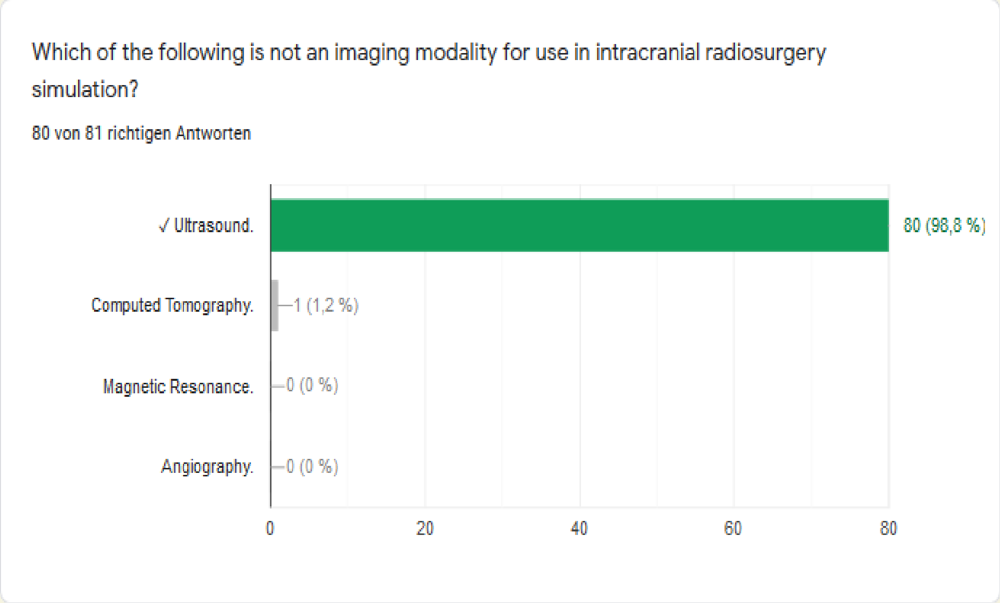

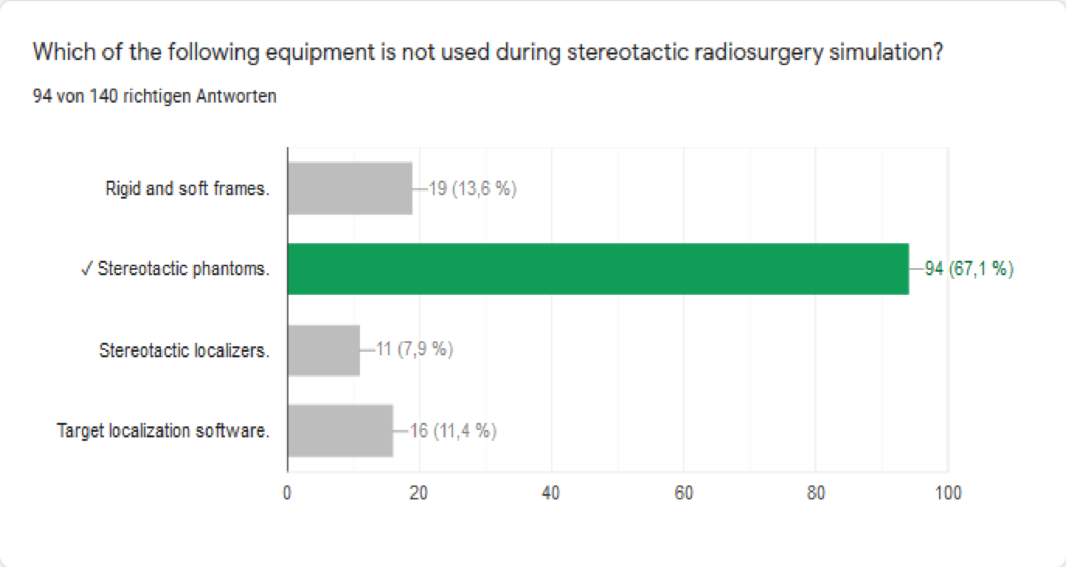

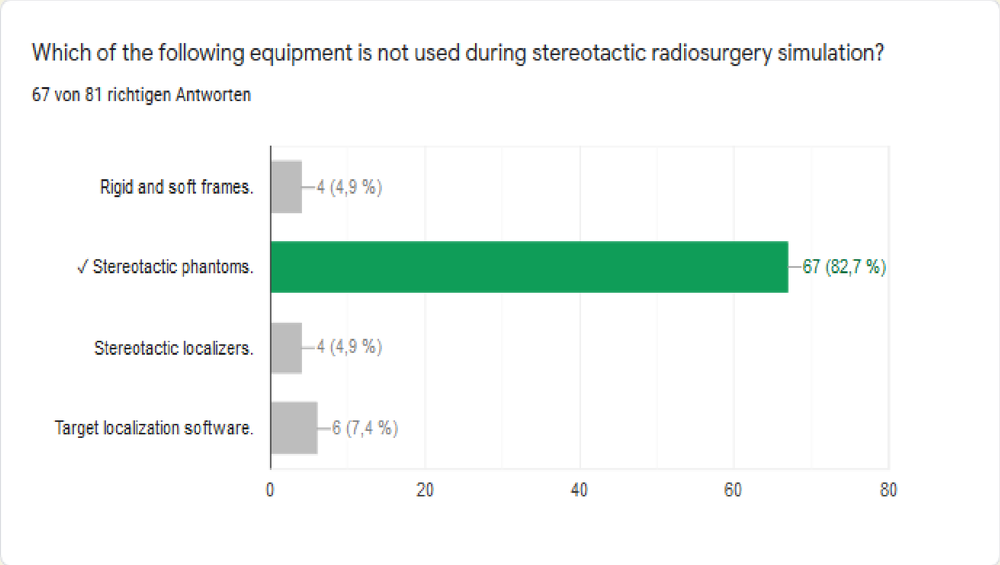

Pre- (left) and post-curriculum (right) evaluations

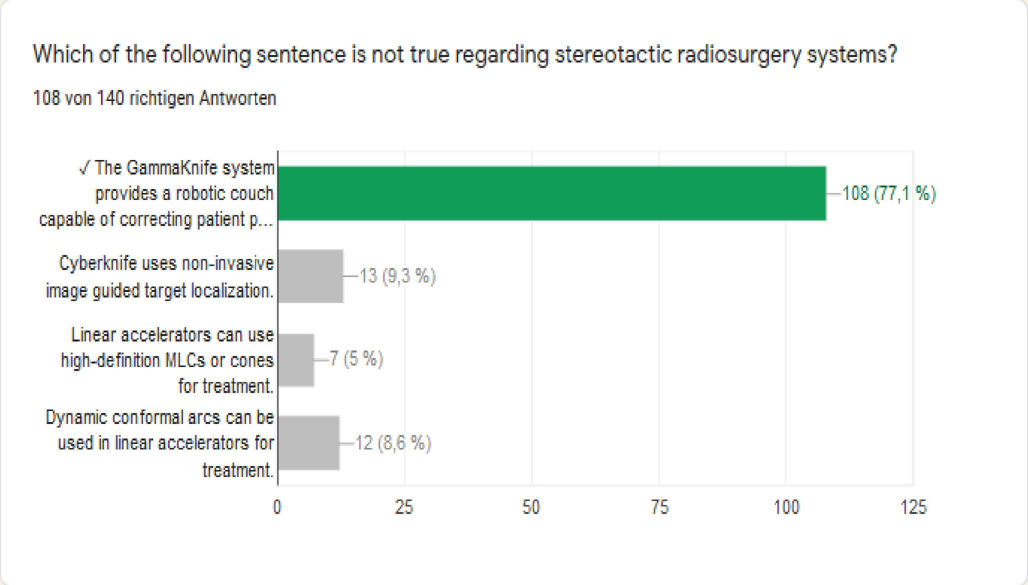

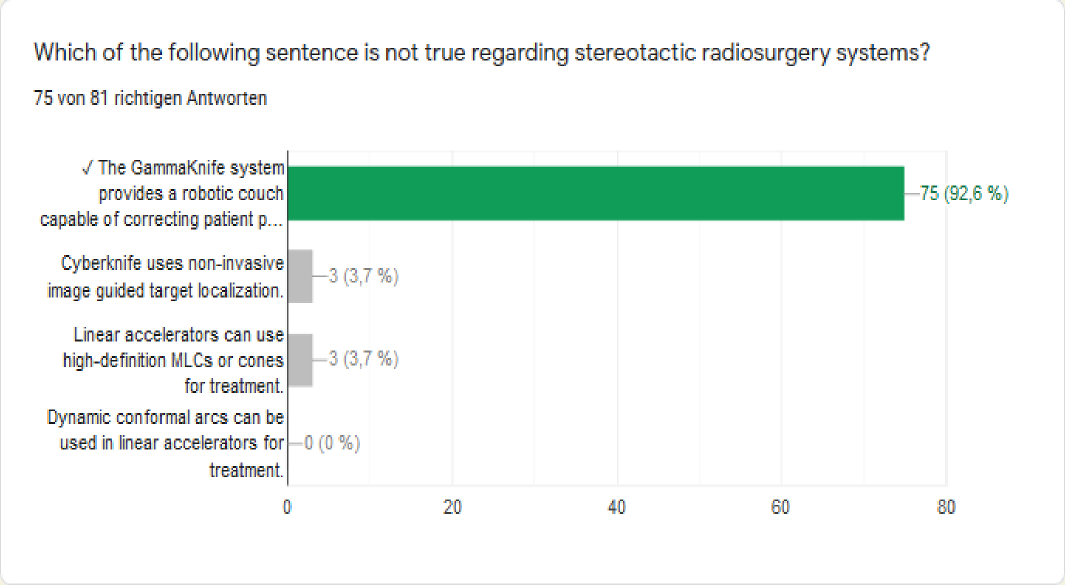

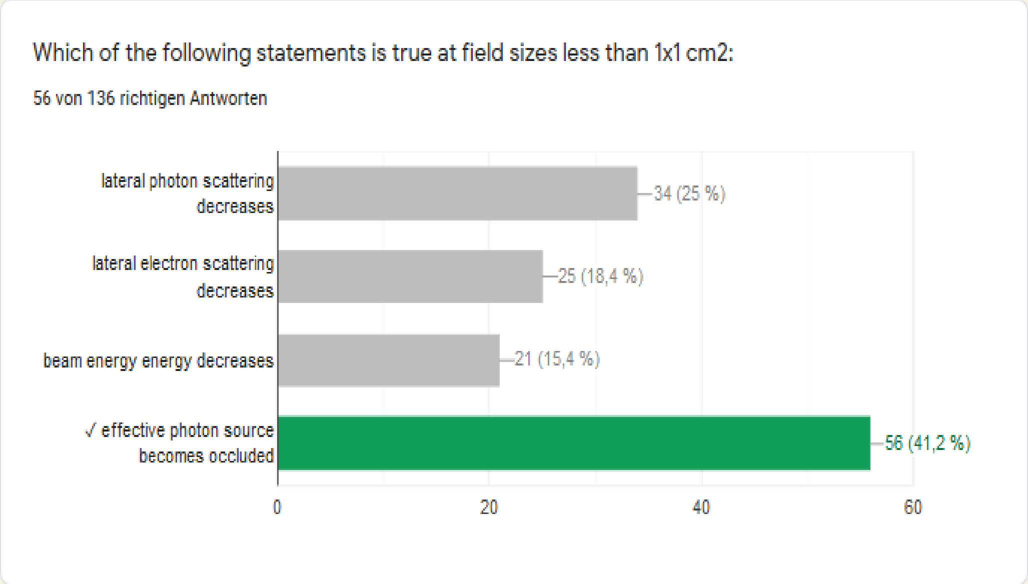

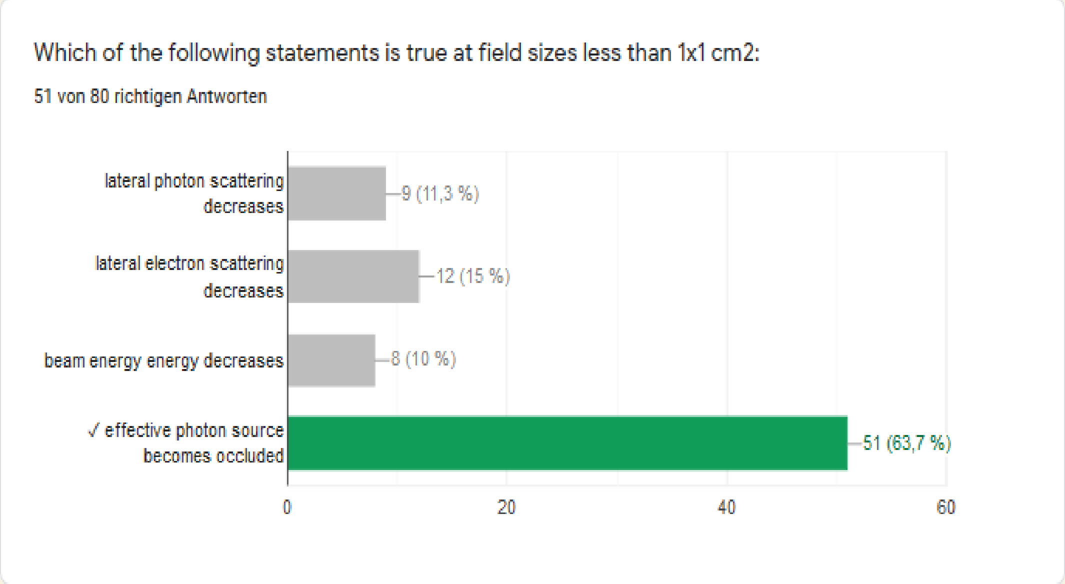

Pre- (left) and post-curriculum (right) evaluations

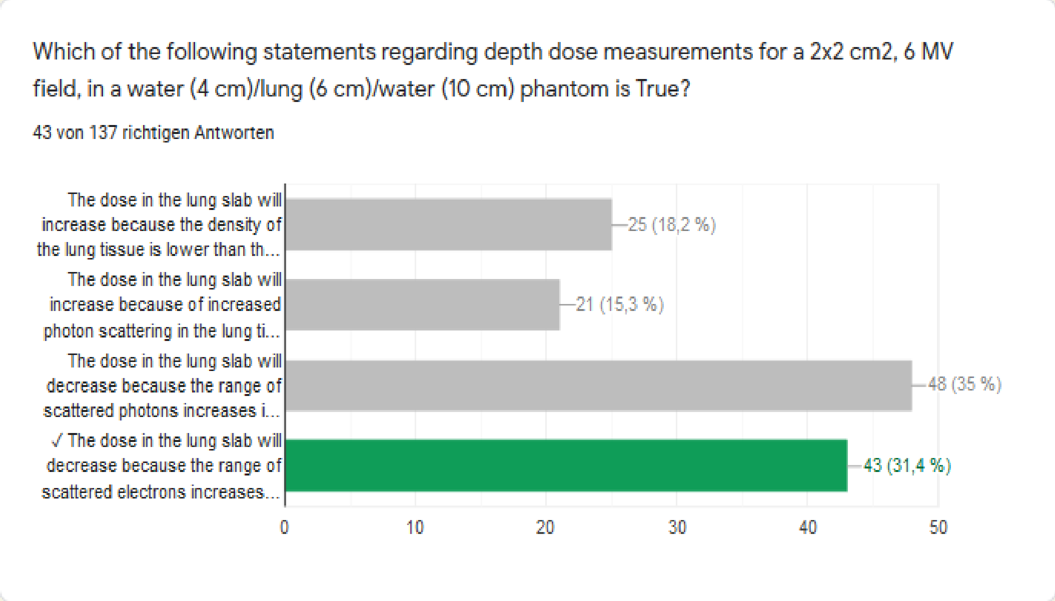

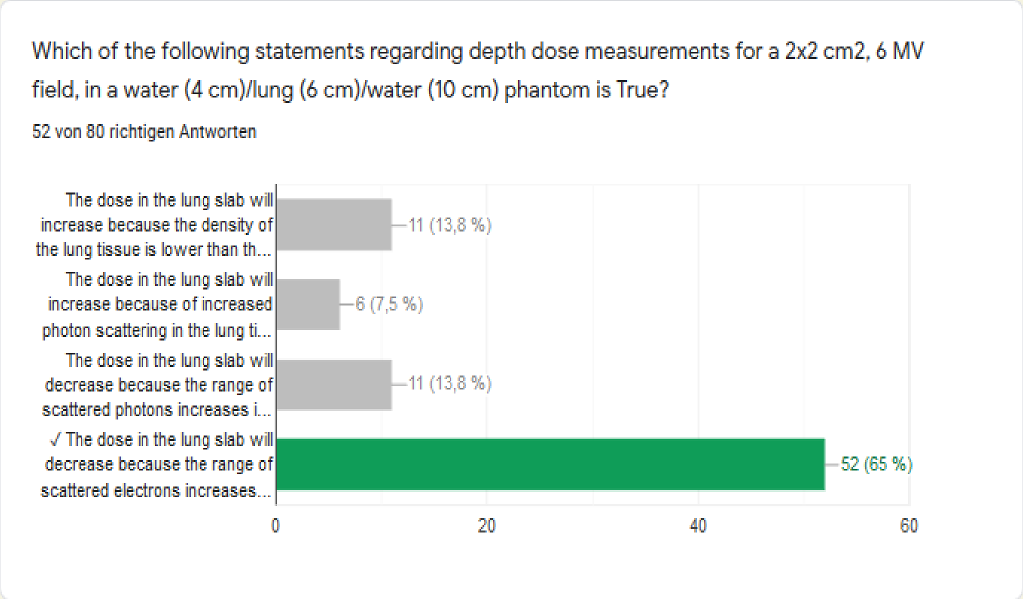

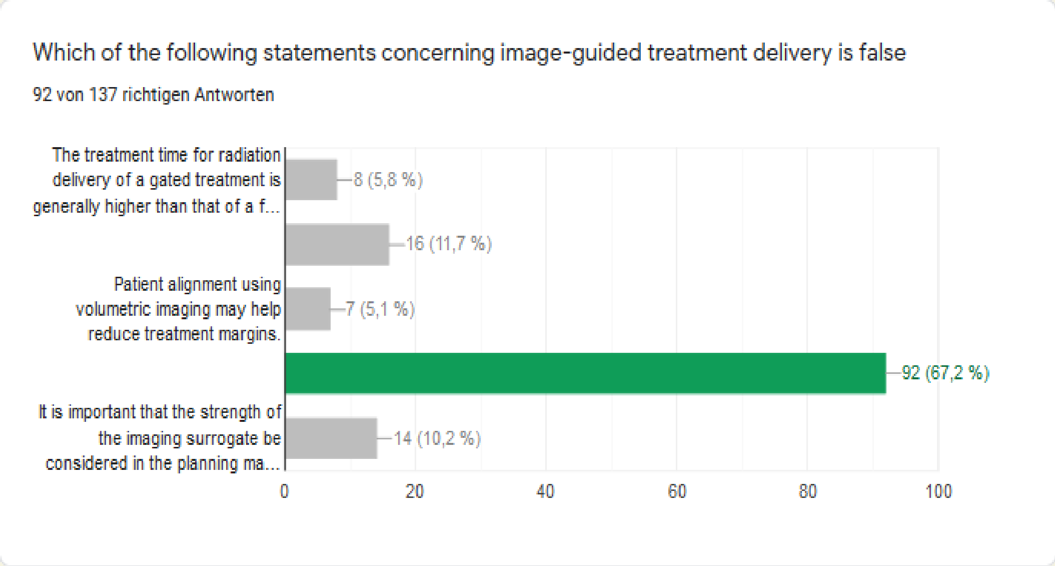

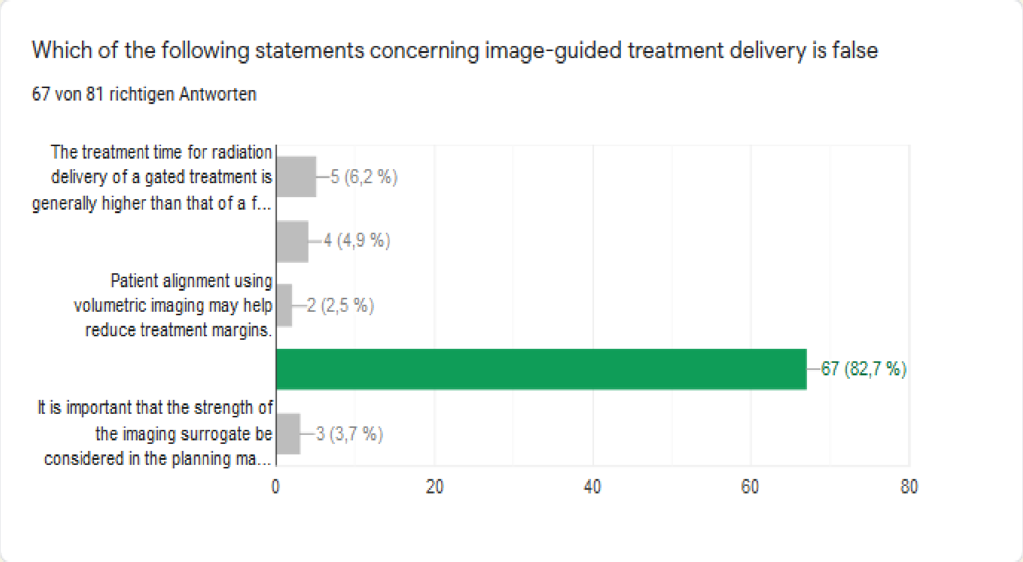

Pre- (left) and post-curriculum (right) evaluations

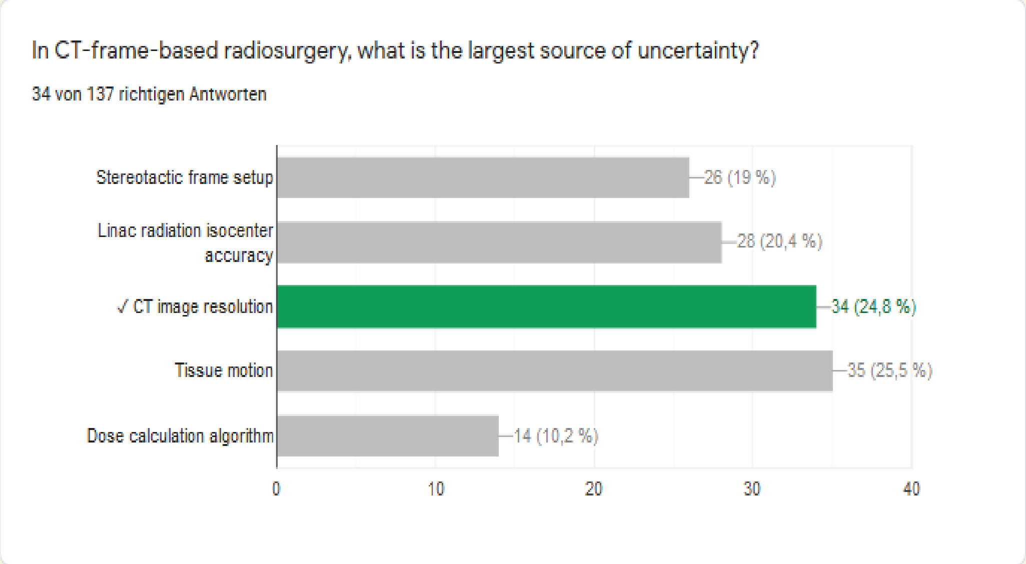

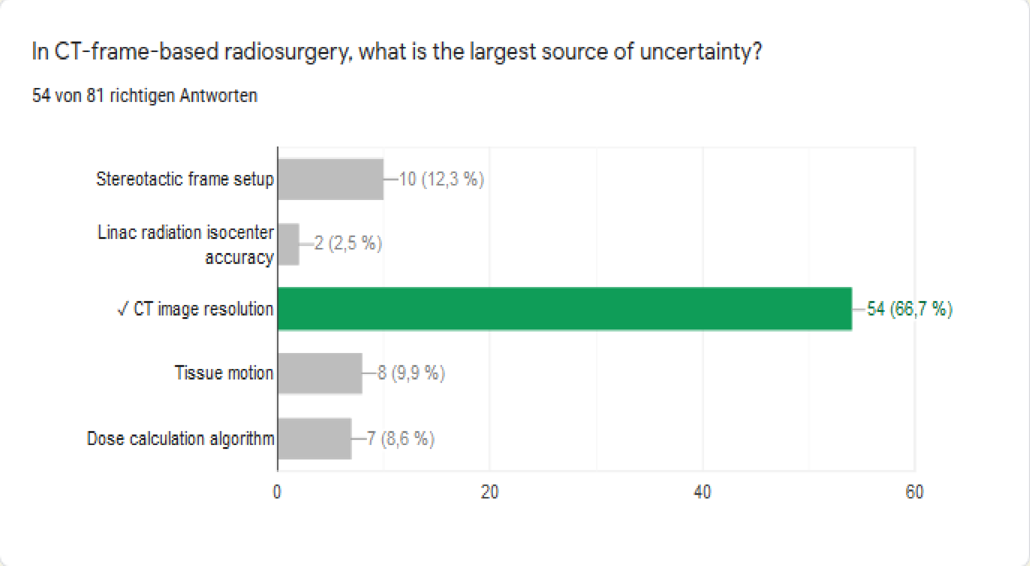

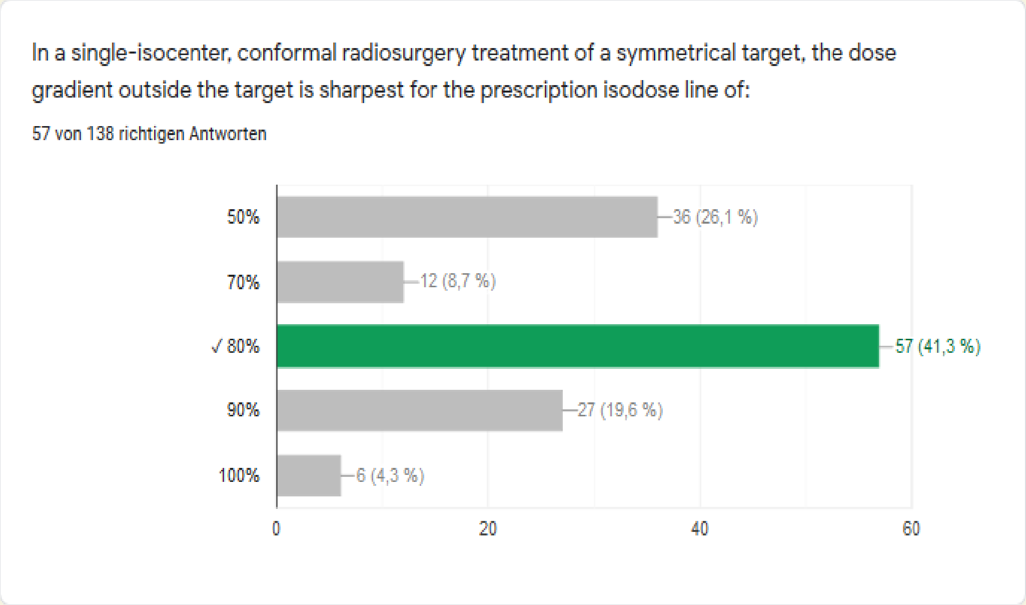

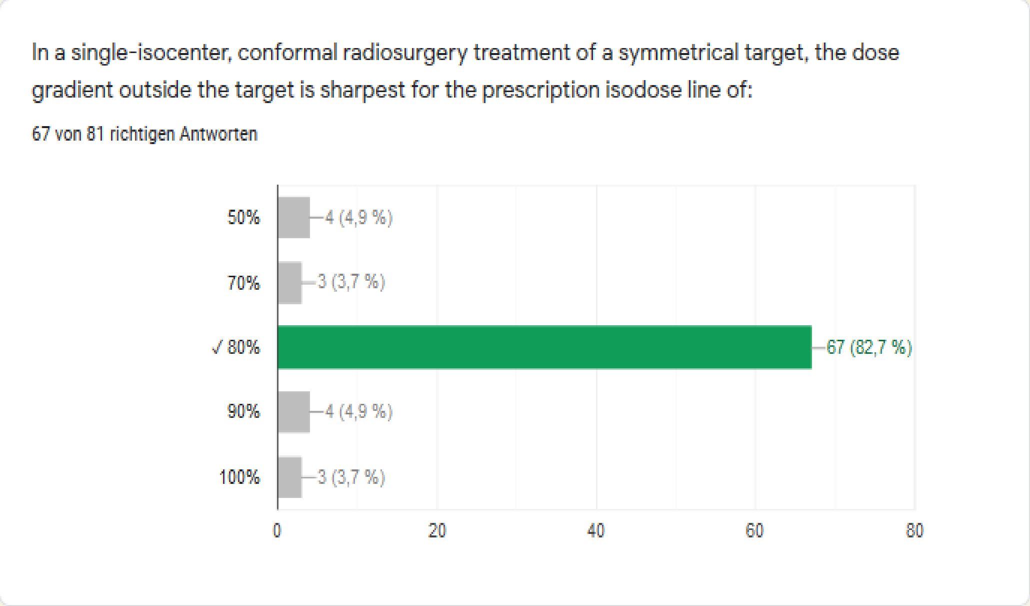

Pre- (left) and post-curriculum (right) evaluations

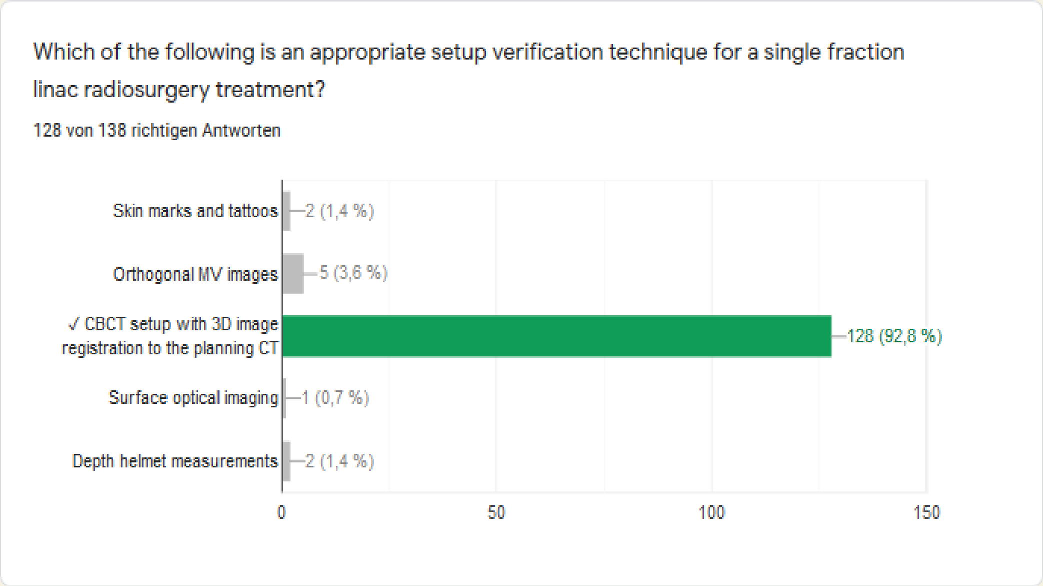

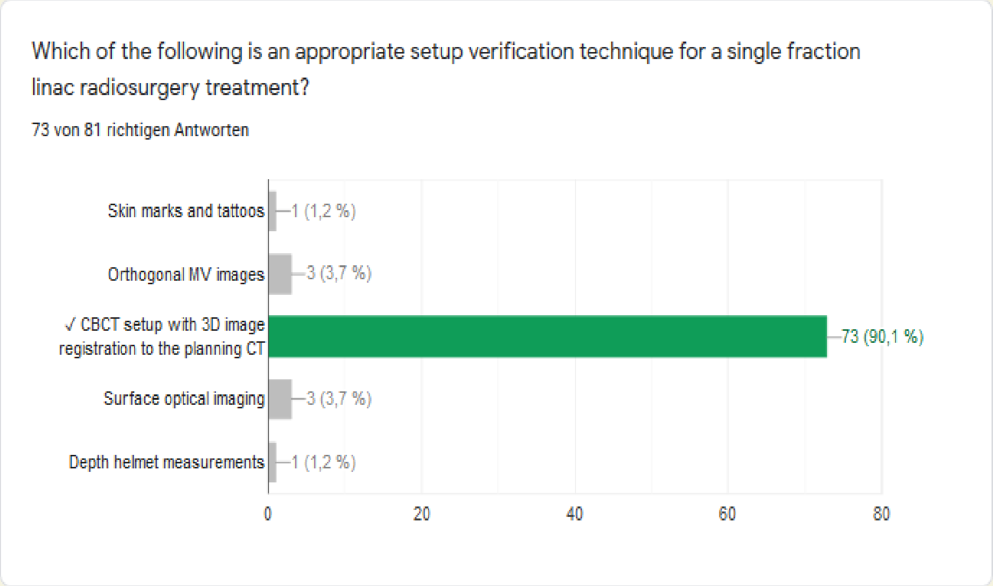

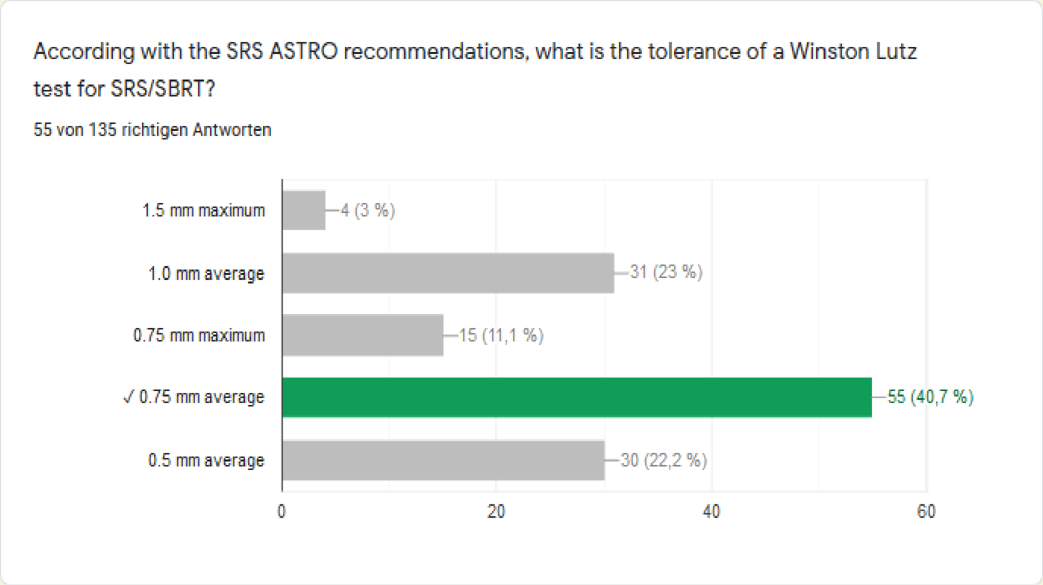

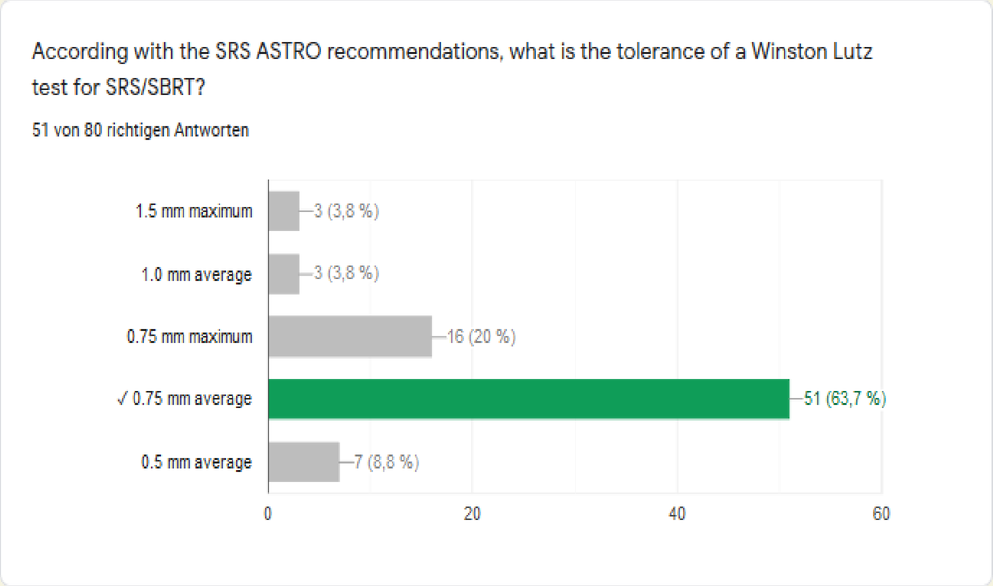

Pre- (left) and post-curriculum (right) evaluations

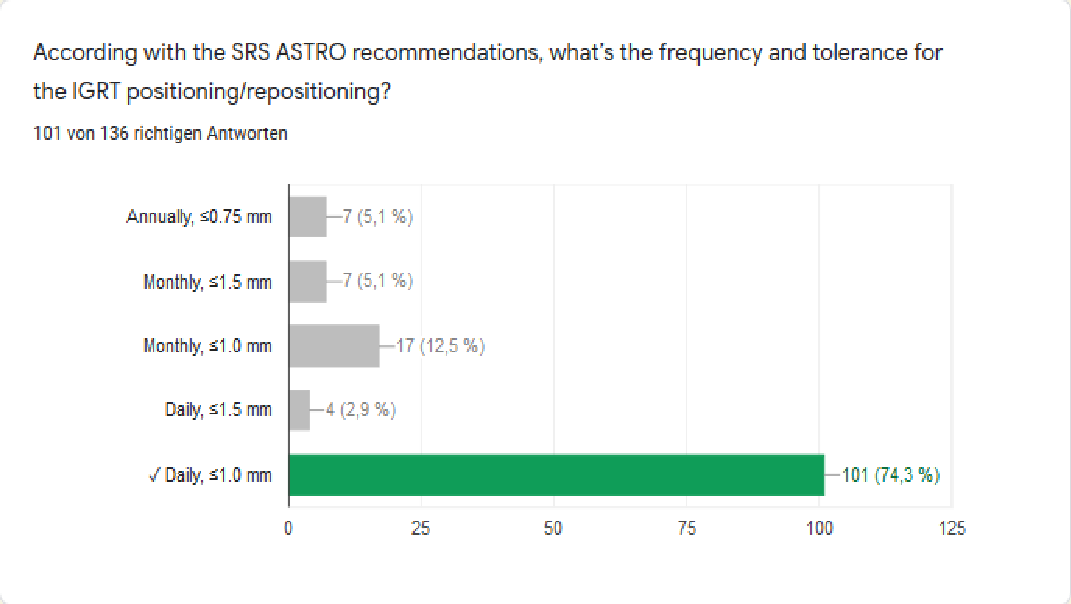

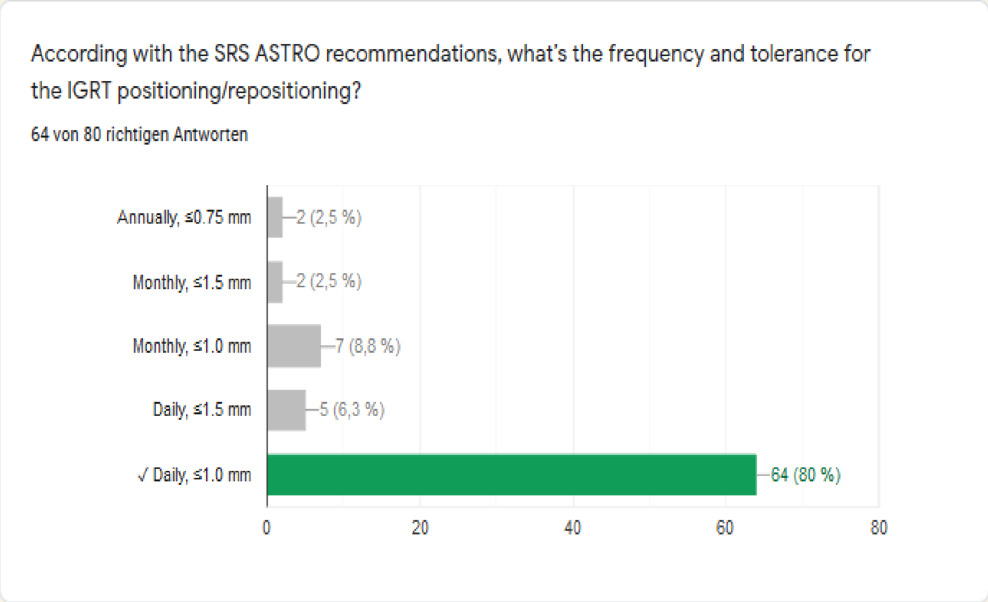

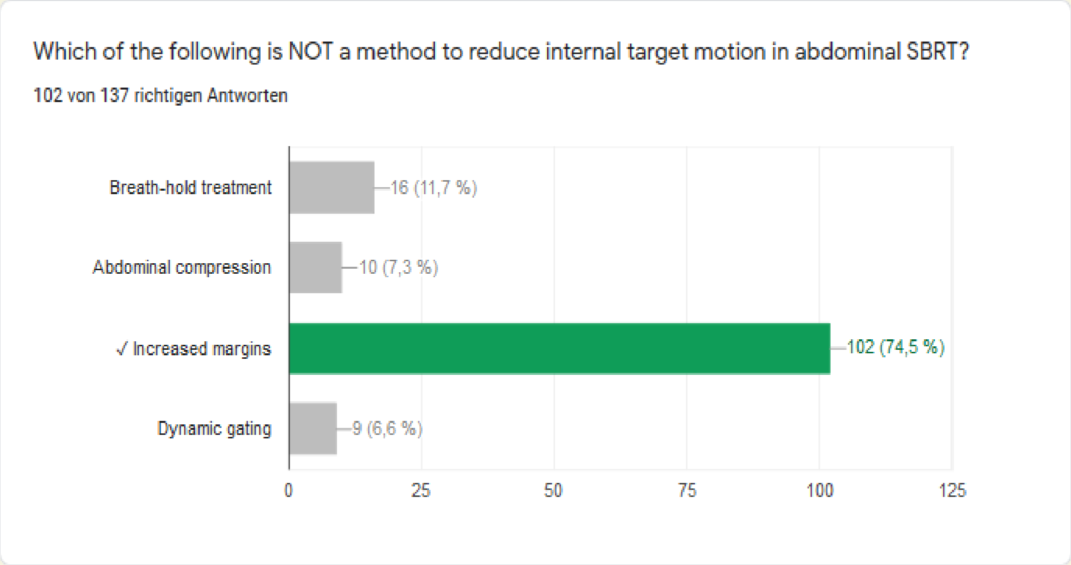

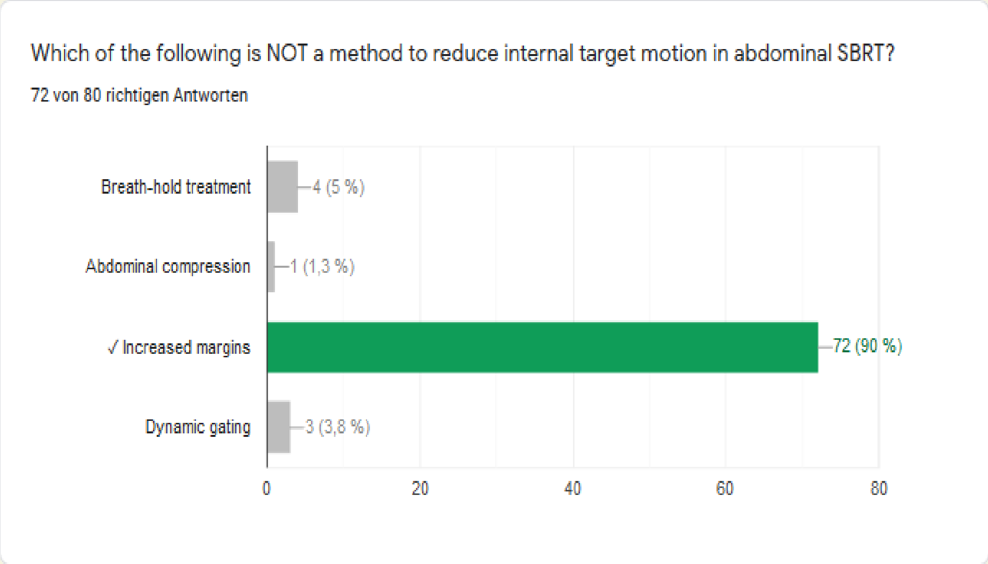

Pre- (left) and post-curriculum (right) evaluations

Accurate target localization at time of treatment is best achieved through which pre-treatment imaging technique?

124 von 137 richtigen Antworten

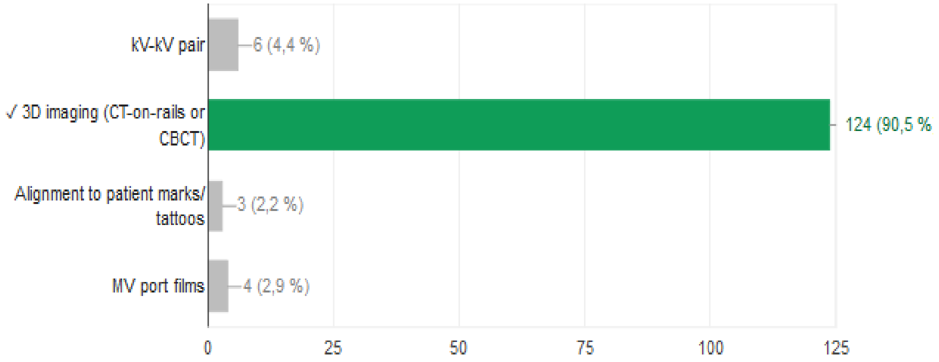

Accurate target localization at time of treatment is best achieved through which pre-treatment imaging technique?

77 von 81 richtigen Antworten

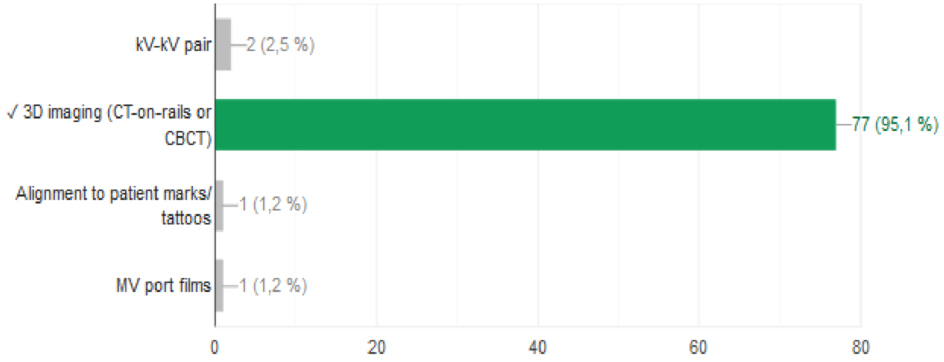

Which one is the gold standard tumor tracking in Radiotherapy?

116 von 137 richtigen Antworten

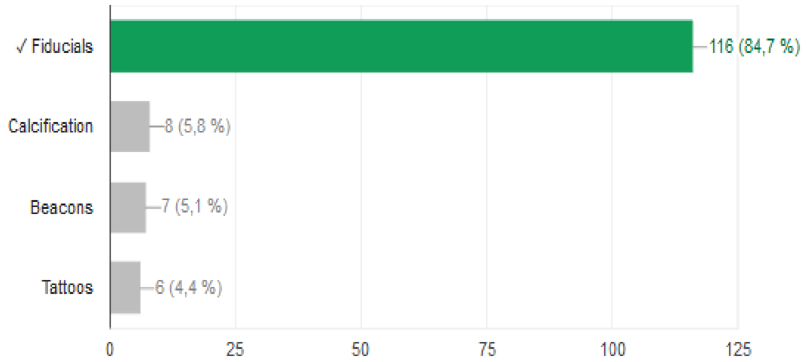

Which one is the gold standard tumor tracking in Radiotherapy?

77 von 81 richtigen Antworten

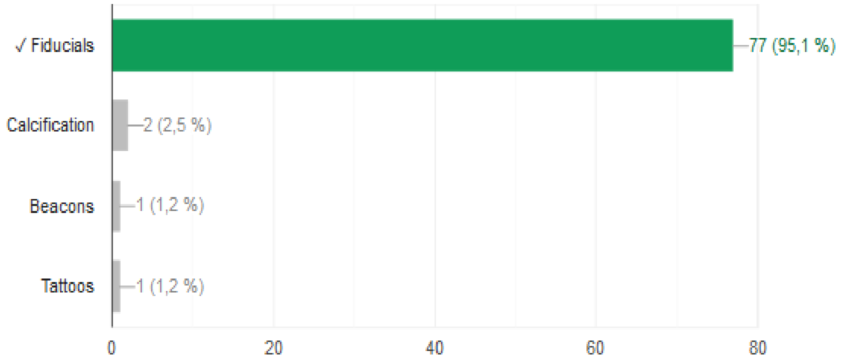

Pre- (left) and post-curriculum (right) evaluations

Which tumor tracking method is used by Cyberknife for intracranial lesions?

72 von 136 richtigen Antworten

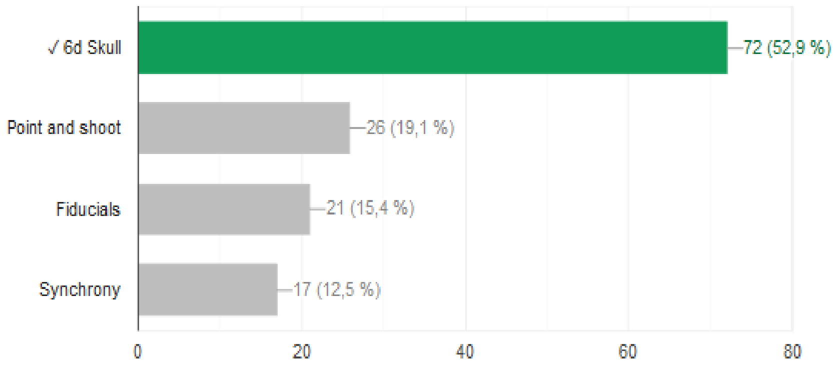

Which tumor tracking method is used by Cyberknife for intracranial lesions?

71 von 81 richtigen Antworten

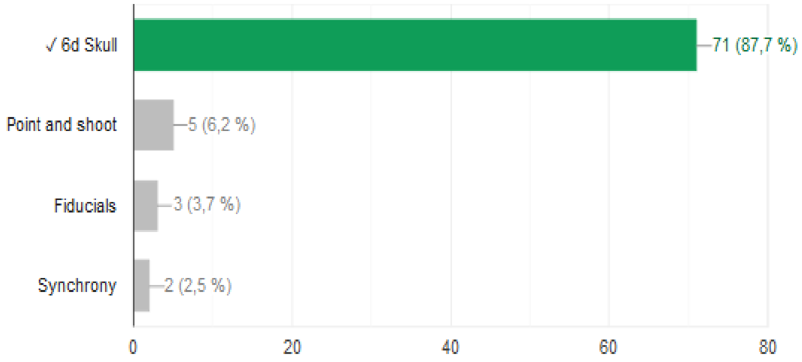

For the daily output (cGy/MU) constancy check, what's the expected deviation from baseline?

64 von 135 richtigen Antworten

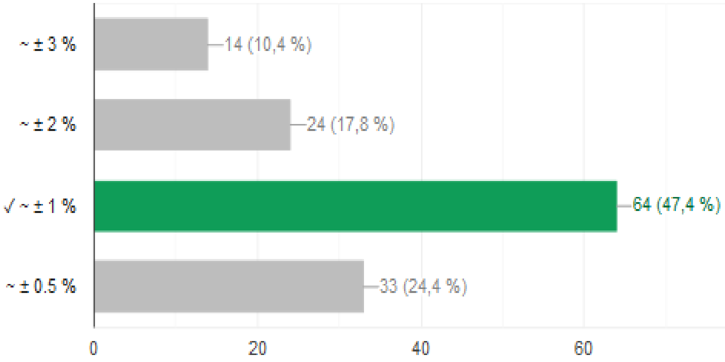

For the daily output (cGy/MU) constancy check, what's the expected deviation from baseline?

69 von 81 richtigen Antworten

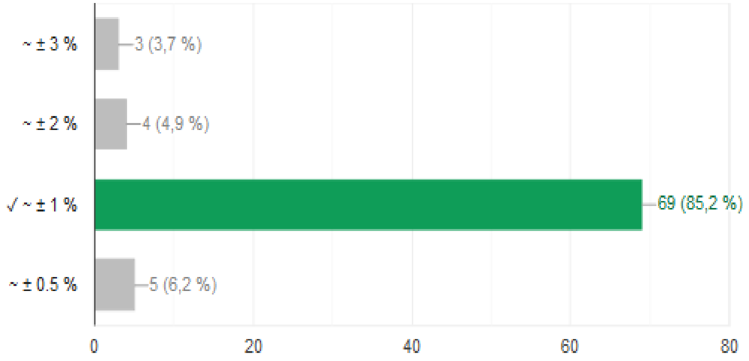

Pre- (left) and post-curriculum (right) evaluations

For the monthly output (cGy/MU) constancy check, what's the expected deviation from baseline?

38 von 135 richtigen Antworten

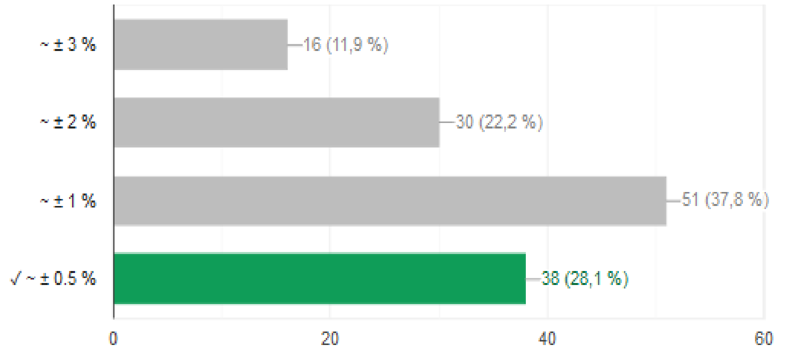

For the monthly output (cGy/MU) constancy check, what's the expected deviation from baseline?

61 von 81 richtigen Antworten

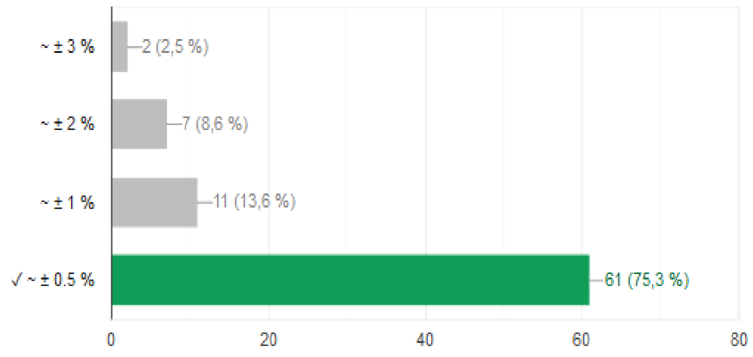

The robotic arm positioning error determined from film measurements should be approximately?

70 von 135 richtigen Antworten

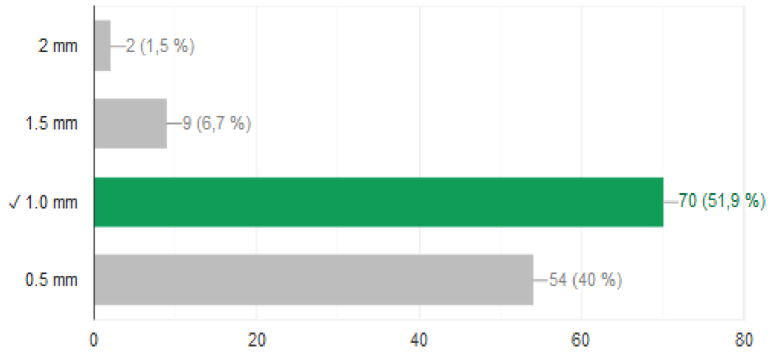

The robotic arm positioning error determined from film measurements should be approximately?

66 von 81 richtigen Antworten

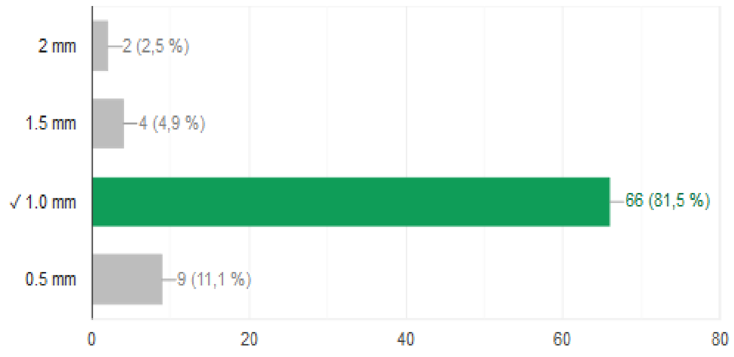

Pre- (left) and post-curriculum (right) evaluations

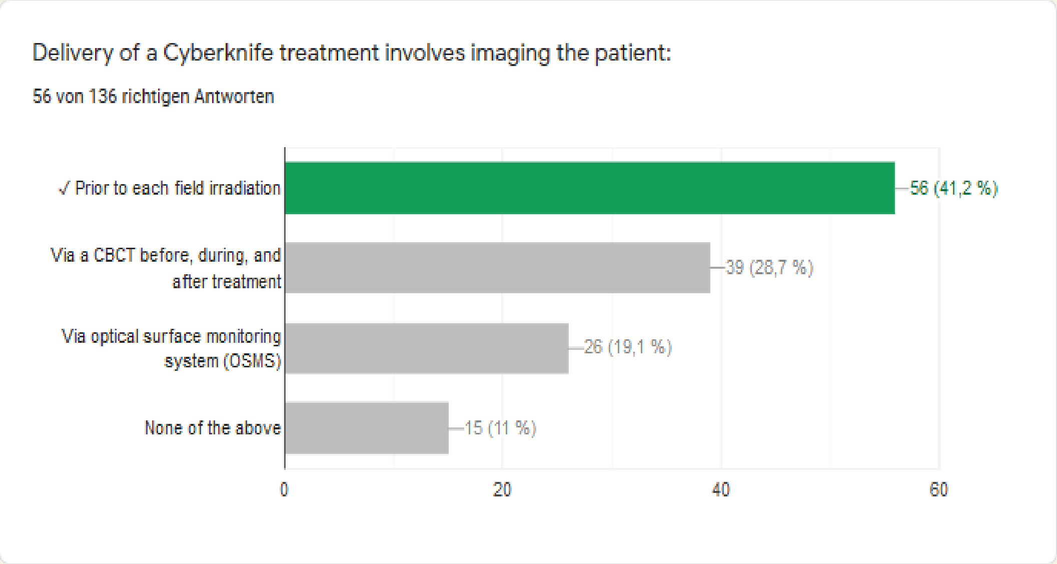

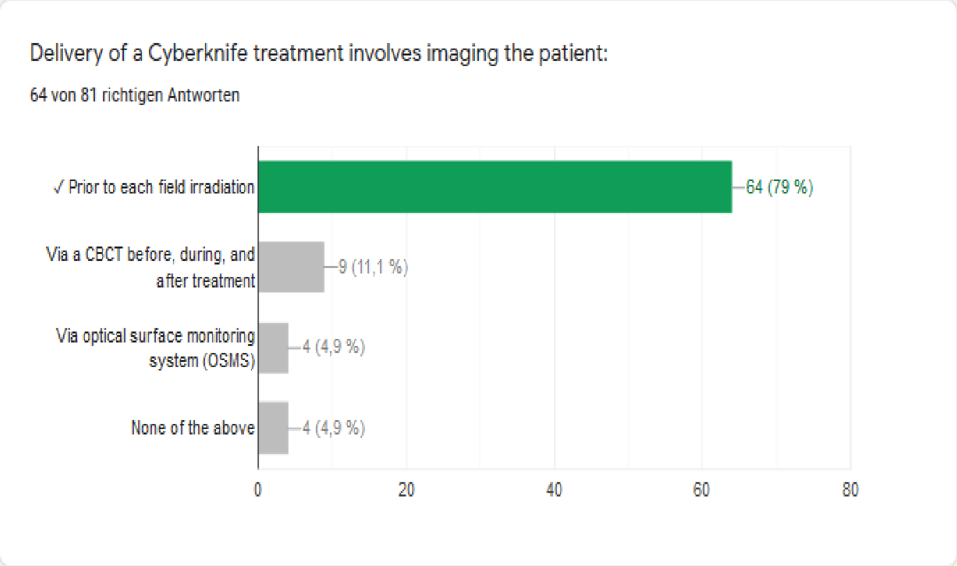

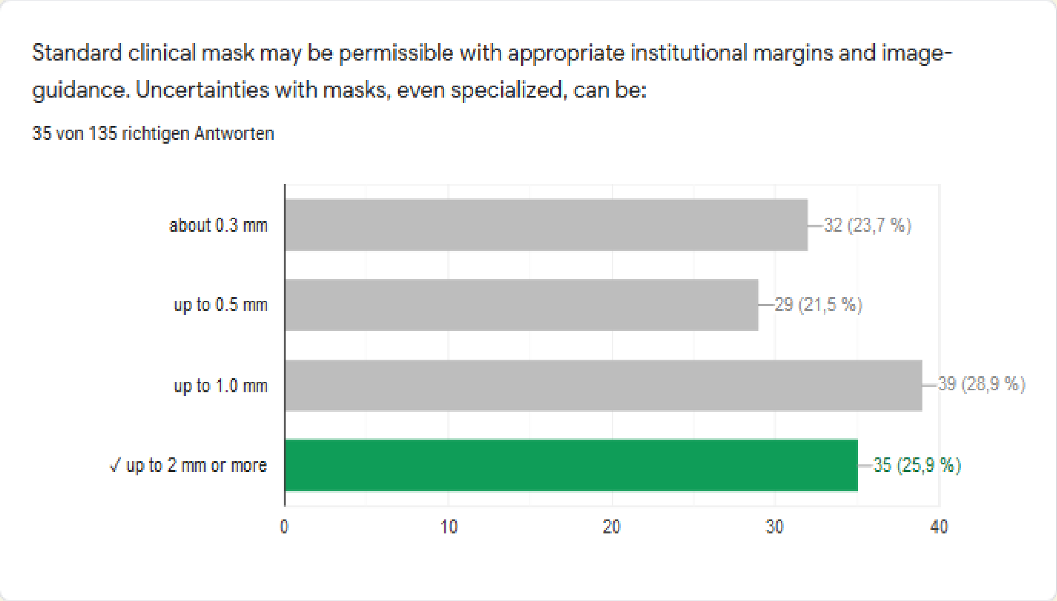

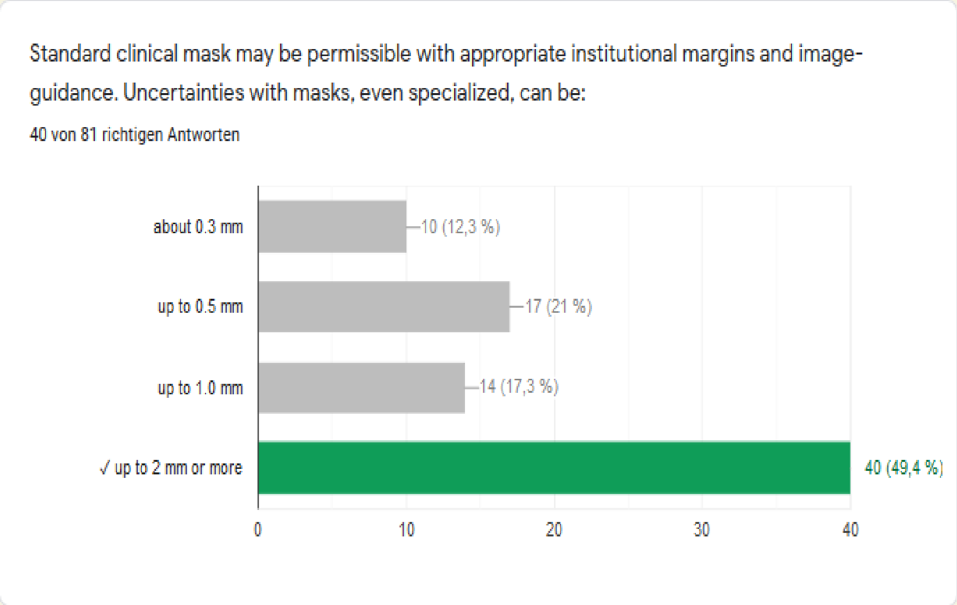

Pre- (left) and post-curriculum (right) evaluations

"Highly Reliable Organizations," have several key traits: sensitivity to operations, reluctancy to accept "simple" explanations for errors, preoccupation with failure, deference to expertise, and resiliency. Which is not an example of such a trait:

68 von 133 richtigen Antworten

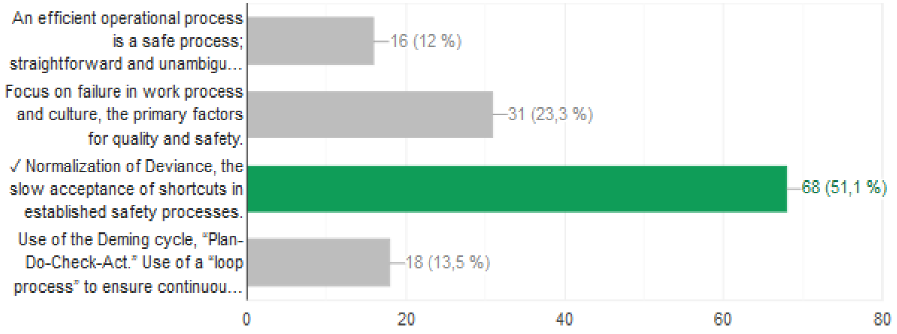

"Highly Reliable Organizations," have several key traits: sensitivity to operations, reluctancy to accept "simple" explanations for errors, preoccupation with failure, deference to expertise, and resiliency. Which is not an example of such a trait:

63 von 81 richtigen Antworten

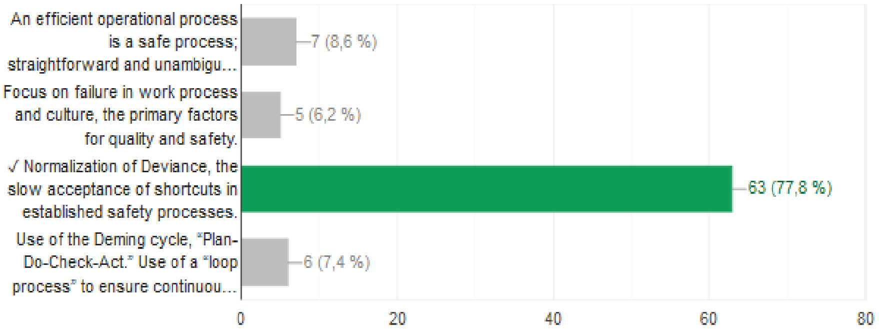

Stereotactic treatments require end-to-end tests on a regular basis, ideally embedded within a well-implemented PDCA (Plan-Do-Check-Act) continuous quality improvement cycle. An end-to-end test is best because:

97 von 135 richtigen Antworten

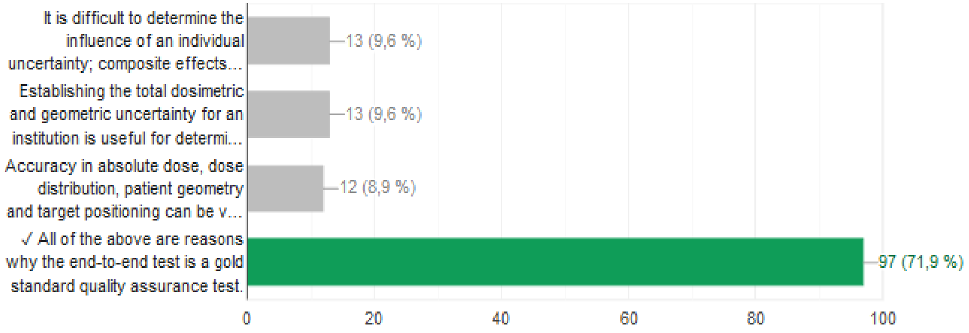

Stereotactic treatments require end-to-end tests on a regular basis, ideally embedded within a well-implemented PDCA (Plan-Do-Check-Act) continuous quality improvement cycle. An end-to-end test is best because:

72 von 81 richtigen Antworten

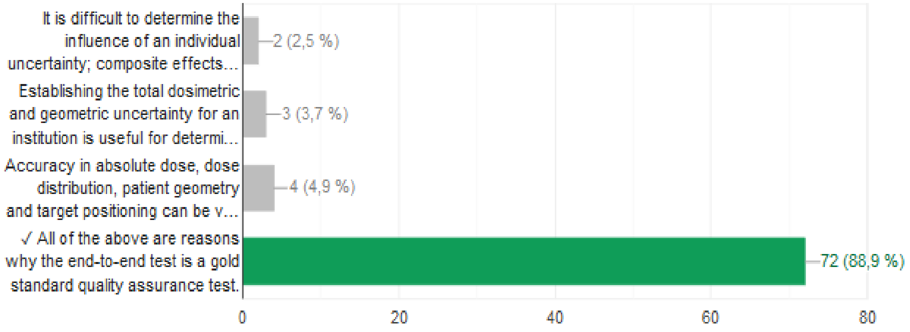

Supplement: Supplementary file 1 [file DataSheet_1.zip › Appendix 3.PDF]
